# Vaginal Microbial Risk Factors Associated with Human Papillomavirus-Induced Cervical Intraepithelial Neoplasia: A Systematic Review

**DOI:** 10.3390/ijms27198625

**Published:** 2026-09-26

**Authors:** Ying Yi Chew, Aastha Sharma, Sheron Sir Loon Goh, Nafeesa Akma Mat Ali, Yolanda Augustin, Sanjeev Krishna, Ivy Chung, Milisha Koh A. Magesvaran, Ayesha Masood, Cindy Shuan Ju Teh

**Affiliations:** 1 Department of Medical Microbiology, Universiti Malaya, Kuala Lumpur 50603, Malaysia; yingyicheww@um.edu.my (Y.Y.C.); aasthasharma@um.edu.my (A.S.); 2 Department of Clinical Pharmacy and Pharmacy Practice, Universiti Malaya, Kuala Lumpur 50603, Malaysia; sherongoh@um.edu.my; 3 School of Health and Medical Sciences, Institute of Infection and Immunity, City St George’s University of London, London EC1V 0HB, UK; nmatali@city.ac.uk (N.A.M.A.); yaugustin@cadt.org.uk (Y.A.); skrishna@cadt.org.uk (S.K.); 4 St George’s University Hospitals NHS Foundation Trust, London EC1V 0HB, UK; 5 Institute of Tropical Medicine, University of Tübingen, Wilhelmstraße 27, 72074 Tübingen, Germany; 6 Centre de Recherches Médicales en Lambaréné (CERMEL), Lambaréné B.P. 242, Gabon; 7 Universiti Malaya Affordable Diagnostics and Therapeutics, Department of Pharmacology, Universiti Malaya, Kuala Lumpur 50603, Malaysia; ivychung@um.edu.my (I.C.); milisha.koh@ummc.edu.my (M.K.A.M.); 8 Department of Public Health Sciences, Health Services Academy, Islamabad 44000, Pakistan; bint12473@gmail.com

**Keywords:** vaginal microbiome, human papillomavirus, cervical intraepithelial neoplasia, community state type

## Abstract

Human papillomavirus (HPV) drives cervical carcinogenesis, but viral infection alone is insufficient for malignant transformation. The vaginal microbiome may modulate HPV persistence and cervical intraepithelial neoplasia (CIN) progression, yet existing reviews remain inconclusive. This systematic review identifies specific vaginal microbiome risk factors associated with HPV and CIN and proposes plausible mechanisms linking dysbiosis to disease progression. Following (PRISMA) guidelines, databases were searched for studies published through October 2025. Thirty-three studies from 2013 to 2025 across 10 countries met the inclusion criteria, with 87.9% exhibiting low risk of bias. Most studies demonstrated marked *Lactobacillus* depletion alongside *Prevotella* and *Gardnerella* enrichment in HPV/CIN cases. Disease severity correlated with shifts toward high-diversity anaerobe communities, though alpha and beta diversity metrics varied across cohorts. Importantly, longitudinal data suggests a temporal sequence where baseline dysbiosis precedes clinical progression. Crucially, although the included cohorts directly observed and quantified these taxonomic shifts, the underlying mechanisms of disease progression, such as enzymatic degradation of mucosal barriers mediated by *Gardnerella* and immunosuppression driven by *Fusobacterium*, remain largely inferred from external in vitro literature. *Lactobacillus iners* emerged as a notable species-level risk factor in HPV persistence. Vaginal dysbiosis, characterised by *Lactobacillus* depletion and anaerobic expansion, is strongly correlated with HPV persistence and CIN progression, potentially facilitated through convergent barrier degradation and immune subversion. These findings highlight targets for microbiome-based risk stratification and therapeutic intervention.

## 1. Introduction

Cervical cancer remains a major global health challenge amongst women worldwide, with high-risk human papillomavirus (HPV) infection recognised as the primary etiological agent [1]. The disease disproportionately affects women in low-income countries, where access to vaccination and screening remains limited [2]. However, HPV infection alone is insufficient to cause malignant transformation. Most infections resolve spontaneously, and only a subset progress through the precancerous stages of cervical intraepithelial neoplasia (CIN) to invasive carcinoma [3]. This suggests that additional co-factors may play a critical role in modulating disease progression.

The vaginal microbiome has increasingly been implicated as a potential co-factor in cervical disease. A healthy vaginal microbiome, dominated by *Lactobacillus* species, maintains a low vaginal pH and protects against pathogens. Based on community state types (CSTs), *Lactobacillus*-dominant niches (CSTs I, II, III, and V) maintain mucosal integrity, whereas CST IV, characterised by a high-diversity, *Lactobacillus*-depleted state, is associated with dysbiosis and increased susceptibility to HPV [4]. Several cohort studies suggest that vaginal dysbiosis may further promote HPV persistence and CIN progression through certain microbial risk factors [5,6]. However, the specific microbial taxa and community profiles associated with HPV and CIN remain poorly defined.

While previous systematic reviews have examined aspects of this relationship, their scope has been limited. Wang et al. (2019) focused solely on *Lactobacillus* species and did not account for other taxa implicated in dysbiosis [4]. Wu et al. (2022) synthesised diversity and taxonomic findings across HPV-associated diseases, but did not systematically link these compositional shifts to the underlying biological mechanisms facilitating disease progression [5]. More recently, Mancilla et al. (2024) restricted their analysis to the Latina population, limiting generalisability to other diverse populations [6]. These reviews lack concurrent mapping of microbial risk taxa and the biological mechanisms linking vaginal dysbiosis to HPV persistence and CIN progression. This systematic review aims to identify microbial risk factors within the vaginal microbiome associated with HPV and CIN, and to clarify the biological mechanisms linking vaginal dysbiosis to cervical disease progression.

## 2. Materials and Methods

This systematic review was registered on PROSPERO (Registration ID: CRD420251167829) and conducted following the Preferred Reporting Items for Systematic Reviews and Meta-Analyses (PRISMA) 2020 guidelines [7].

### 2.1. Data Sources and Search Strategy

The systematic search was conducted across five electronic databases, including PubMed, Web of Science, Scopus, CINAHL, and ProQuest, to retrieve studies published up to October 2025. A combination of Medical Subject Headings (MeSH) terms and free-text keywords was used across three concept domains: vaginal microbiome, HPV, and CIN. Additional studies were identified through backward citation searching of eligible articles and relevant reviews.

### 2.2. Eligibility Criteria

Studies were included if they involved women who were HPV-positive and/or diagnosed with any grade of CIN, with or without comparison or control groups. Eligible studies must have assessed vaginal microbiome composition or explored mechanisms by which the microbiome might influence HPV persistence and CIN development. Only peer-reviewed, full-text articles published in English were included. Doctoral theses, dissertations and preprints were not eligible; where a peer-reviewed article derived from a thesis existed, the article was used as the data source. Studies that identified taxa by culture were eligible if organisms were resolved to genus or species level.

Studies conducted in animals, pregnant women, or women with high-risk co-infections known to independently alter the vaginal microbiome or local immune response (e.g., HIV, HSV-2, or other non-HPV sexually transmitted infections), or on non-cervicovaginal microbiomes (e.g., gut, skin, or anal microbiomes), were excluded. Additionally, studies lacking taxonomic resolution and publications in the form of abstracts, editorials, commentaries, or reviews were not eligible for inclusion.

### 2.3. Study Selection

All records retrieved from database searches were imported into Rayyan AI ((Rayyan Systems Inc., Cambridge, MA, USA, https://www.rayyan.ai/ (accessed on 20 November 2025)) where duplicates were identified and removed before screening. Two reviewers independently evaluated all titles and abstracts against the eligibility criteria, followed by independent full-text assessment of all potentially relevant articles. Discrepancies arising at either stage were resolved through discussion between the two reviewers or, where consensus could not be reached, by involving a third reviewer.

### 2.4. Data Extraction

Data were extracted independently by two reviewers using a standardised extraction form covering the following domains: study characteristics (author, year, design, country, and sample size); participant demographics (age); clinical characteristics (HPV genotype, HPV status, and CIN grade); microbiome data (sequencing method, taxa findings, CST distribution, and alpha and beta diversity); functional and metabolic data (vaginal pH, lactate levels, enzymatic activity, metabolomics, and immune response). Mechanistic actions were extracted from the authors’ discussions and mainly include external in vitro literature for context, rather than reflecting primary in vivo findings from the cohorts. Each mechanistic statement was classified by evidence tier: tested (measured in the study’s own samples, for example an enzyme assay, metabolomics, cytokine assay or pH), inferred (derived from taxonomic or metagenomic data, predicted function, or statistical mediation), or proposed (taken from external literature or the authors’ discussion and not measured in the cohort).

### 2.5. Quality Assessment

The methodological quality of all included studies was appraised independently by two reviewers using the Joanna Briggs Institute (JBI) critical appraisal tools. Analytical cross-sectional studies were assessed using the JBI Checklist for Analytical Cross-Sectional Studies, while cohort studies were evaluated using the JBI Checklist for Cohort Studies [8]. Each item was rated as yes, no, unclear, or not applicable. Any disagreements between reviewers were resolved through consultation with a third reviewer.

### 2.6. Outcome and Analysis

The primary outcome of this review was to identify microbial taxa and CSTs associated with HPV infection and CIN, while the secondary outcome focused on the biological mechanisms linking vaginal dysbiosis to HPV persistence and CIN progression, including changes in vaginal pH, metabolite profiles, enzymatic activity, and host immune response.

Due to the heterogeneity in sequencing platforms, bioinformatics pipelines, and study populations, a meta-analysis and formal sensitivity analyses were not conducted, and findings were analysed and reported narratively. Given the methodological and clinical heterogeneity across included studies, a formal quantitative certainty of evidence assessment (e.g., GRADE) was not conducted. Instead, the overall robustness of the evidence was assessed through the risk-of-bias appraisal and the biological consistency of the narrative synthesis. Alpha diversity (species richness and evenness within a sample) and beta diversity (compositional differences between groups) were examined to determine whether HPV-positive women or those with higher CIN grades differed from controls. Taxonomic findings were reported across all levels (phylum to species), with particular attention to CST distribution, especially the prevalence of CST IV, which is characterised by low *Lactobacillus* dominance and high microbial diversity, compared with *Lactobacillus*-dominant CSTs (I, II, III, and V), across HPV and CIN groups. Functional and metabolic findings were also summarised to support mechanistic interpretation. Directional taxon findings were tallied as the number of studies reporting a genus as higher or lower in HPV-positive or CIN groups than in controls; species-level findings were assigned to their genus. These tallies are descriptive. They are not weighted by sample size, effect size or risk of bias, and studies that may share participants were flagged. For alpha and beta diversity, each study was classified as significant (all reported indices or comparisons significant), partially significant (some but not all), not significant, or not reported. Test statistics, *p*-values and effect sizes were extracted where available.

Data visualisation was performed in R version 4.6.1 (R Foundation for Statistical Computing, Vienna, Austria) [9] within RStudio (version 2026.06.0, Posit Software, PBC) [10], using the ggplot2 package (Posit Software, PBC) [11]. A qualitative colour palette for taxa was generated using the Polychrome package [12] to ensure visual distinction across genera.

## 3. Results

**Figure 1:**
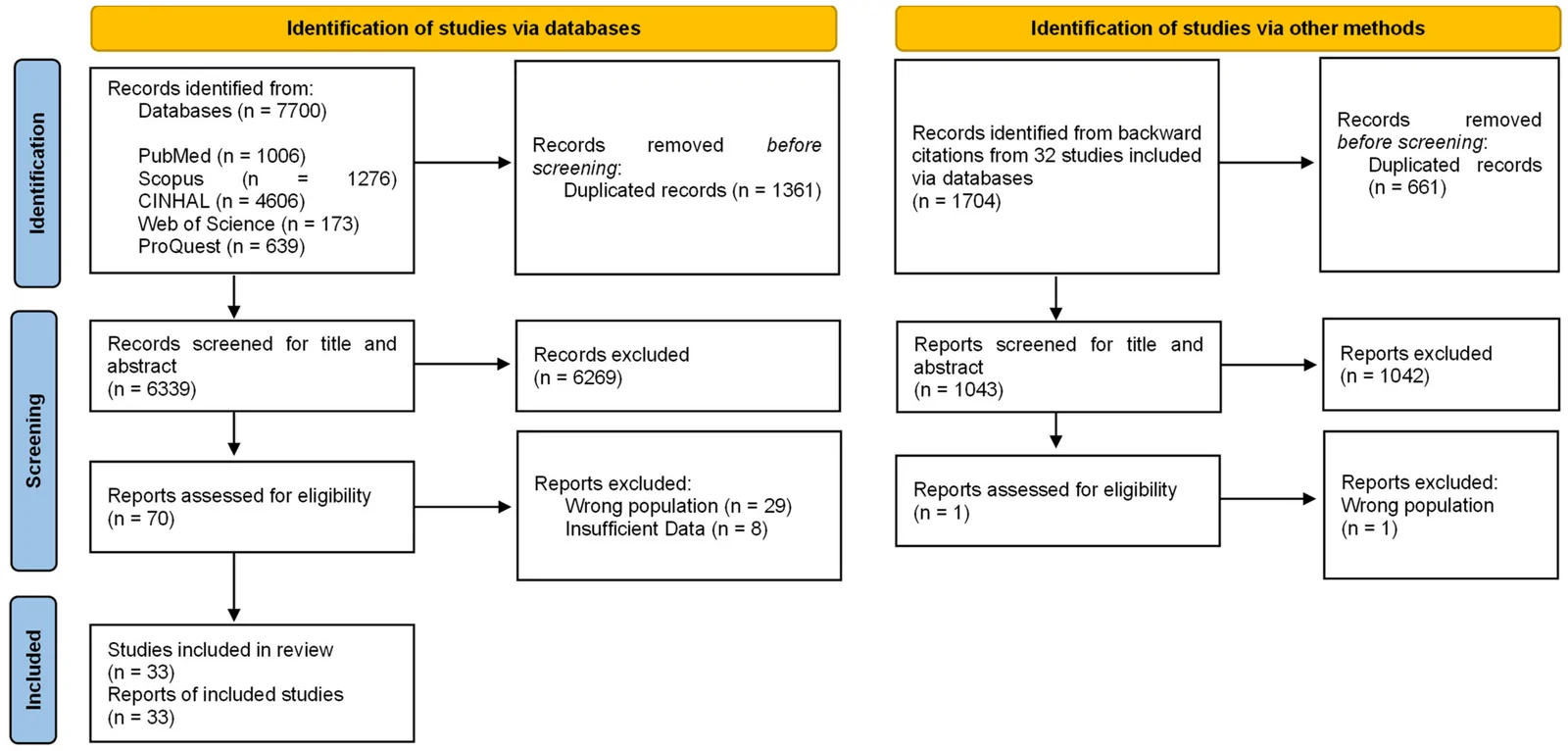
PRISMA diagram for identification and selection of studies.

A total of 27 cross-sectional [13,14,15,16,17,18,19,20,21,22,23,24,25,26,27,28,29,30,31,32,33,34,35,36,37,38,39] and 6 longitudinal studies [40,41,42,43,44,45] met our inclusion criteria (Figure 1). The PRISMA 2020 checklist is presented in Table S1, and the search strategy is presented in Table S2.

### 3.1. General Characteristics of Included Studies

The included studies were published between 2013 and 2025 across ten countries, with over half (*n* = 17, 52%) originating in China alone [13,14,15,17,18,19,20,21,27,28,29,33,36,38,39,42,45] (Table 1). The remaining 15 studies were distributed across Asia (South Korea [23,25,26,35], *n* = 4; Taiwan [37], *n* = 1), Europe (Sweden [16,32,40], *n* = 3; United Kingdom [30], *n* = 1; Italy [34], *n* = 1; Russia [24], *n* = 1), North America (USA [22,41,43], *n* = 3), and South America (Brazil [31], *n* = 1; Costa Rica [44], *n* = 1). No studies from Africa or the Indian subcontinent were identified, underscoring significant geographic gaps in the evidence base despite the substantial burden of cervical cancer in these regions. Comparison groups ranged from simple HPV-positive/negative contrasts to multi-tiered designs spanning the full disease spectrum. Reported mean ages generally ranged from the early 30s to 40s. HPV detection and microbiome sequencing methods were heterogeneous: most studies used Polymerase Chain Reaction (PCR)-based HPV assays and 16S ribosomal RNA (rRNA)/rDNA amplicon sequencing (commonly V3–V4, Illumina platforms), with smaller subsets using pyrosequencing [25], shotgun metagenomics [23,32] or metabolomics [22].

### 3.2. Quality Assessment

The Joanna Briggs Institute (JBI) critical appraisal checklists were used to evaluate the studies, focusing on cross-sectional (8 items; *n* = 27) and cohort designs (11 items; *n* = 6). Item-level ratings and scores are in Table S3. Studies meeting at least 70% of applicable items were rated low-risk, while those meeting 50–69% were rated moderate-risk. Confounding was the main weakness. Only 13 of 33 studies (39%) [16,17,20,22,24,28,31,32,39,41,42,43,44] both identified confounders and stated how they were handled, whereas 16 of 29 low-risk studies (55%) [13,14,15,18,19,23,25,26,29,33,34,35,36,37,38,40] did not meet both items. Despite this, most studies (87.9%, *n* = 29) [13,14,15,16,17,18,19,20,22,23,24,25,26,28,29,31,32,33,34,35,36,37,38,39,40,41,42,43,44] were rated low-risk, indicating good methodological quality across the included evidence, whereas four studies were rated as having moderate risk [21,27,30,45]. Importantly, while 87.9% of studies achieved a “low risk” designation, a high checklist score reflects comprehensive reporting rather than the elimination of bias. These observational findings warrant cautious interpretation; high scores do not eliminate the substantial risk of residual confounding, such as unmeasured differences in sexual behaviour, co-infections, or smoking, inherent to cross-sectional study designs.

### 3.3. Microbial Taxa Changes Across HPV and CIN Disease Stages

**Figure 2:**
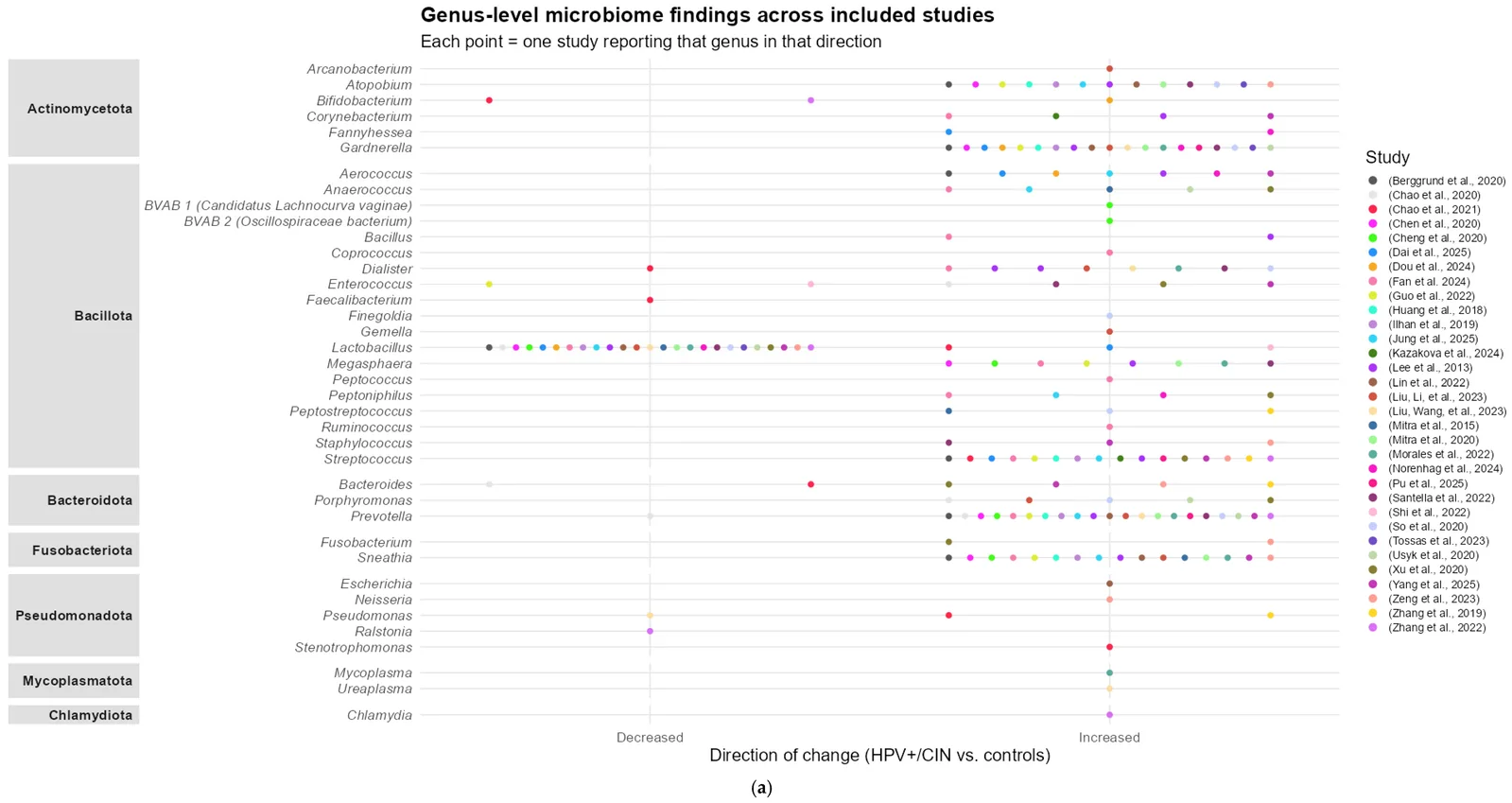

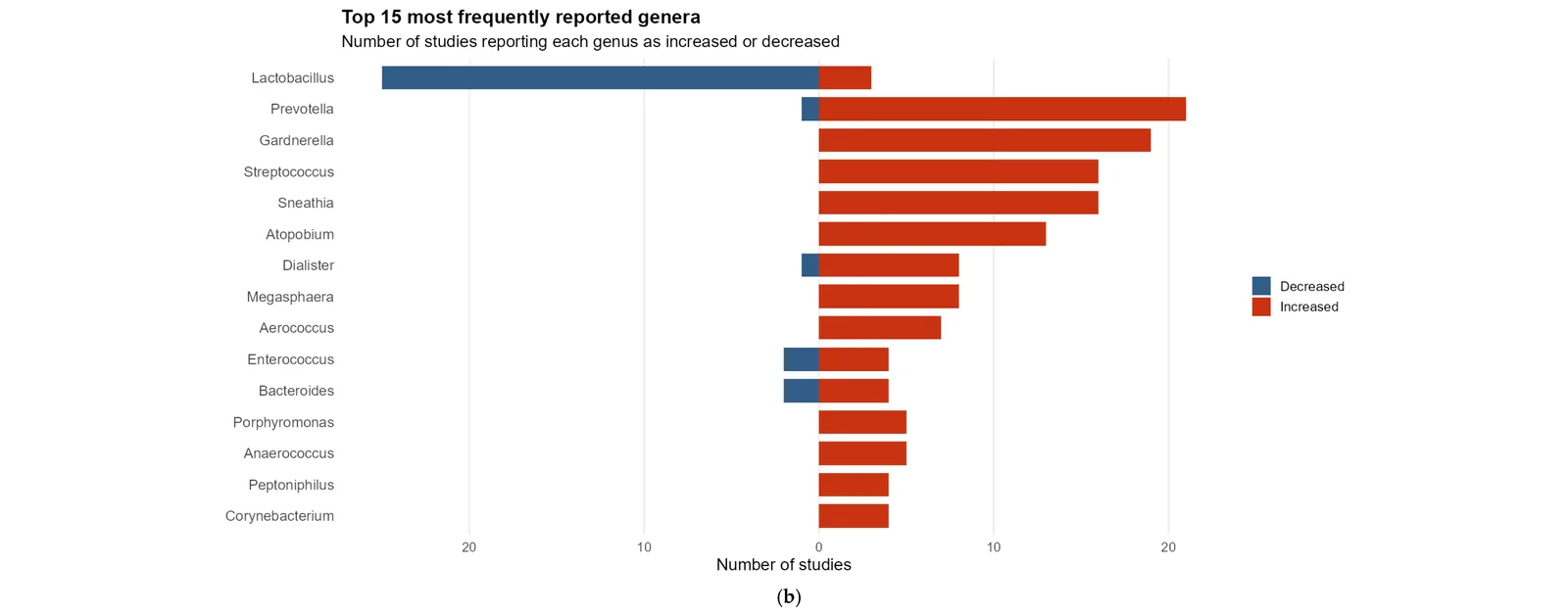
(**a**) Conserved genus-level vaginal microbiome shifts in patients with HPV and CIN. Each point represents one included study reporting a given genus as increased or decreased in HPV-positive/CIN samples relative to controls; genera are grouped by phylum (*Actinomycetota*, *Bacillota*, *Bacteroidota*, *Fusobacteriota, Pseudomonadota*, *Mycoplasmatota*, *Chlamydiota*), and points are coloured by study. Abbreviations: HPV, human papillomavirus; CIN, cervical intraepithelial neoplasia. References cited in this figure: [13,14,15,16,17,18,19,20,21,22,23,24,25,26,27,28,29,30,31,32,33,34,35,36,37,38,39,40,41,42,43,44,45]. (**b**) Direction of change for the top 15 most frequently reported genera across HPV and CIN disease stages. Bars show the number of studies reporting each genus as decreased (blue, left) or increased (red, right). Genera are ordered top to bottom by total number of directional reports.

A highly consistent dysbiotic pattern was observed across disease stages, characterised by depletion of *Lactobacillus* (*n* = 29, 88%) and enrichment of *Prevotella* (*n* = 22, 95%) and *Gardnerella* (*n* = 19, 100%) among women with HPV and CIN (Figure 2a). *Lactobacillus* decreased in most cross-sectional cohorts (24 of 27 reports, 93%), whereas other taxa showed increases. *Prevotella* (21 of 22 reports, 95%) and *Gardnerella* (19 of 19 reports, 100%) were almost always increased in HPV-positive/CIN samples (Figure 2b). This enrichment was observed across *Actinomycetota*, *Bacteroidota*, and *Fusobacteriota.* These counts describe how often a direction was reported. They do not measure the size or reliability of an association.

However, because most of these estimates pool HPV-positive women without lesions together with CIN and cancer cases, we examined the subset of studies that directly compared disease stages to assess whether dysbiosis intensifies progressively or is already established at HPV infection alone. Among the studies that stratified findings by specific cervical lesion grades, a distinct microbial trajectory emerges. Progression from transient HPV infection to low-grade lesions (CIN1/LSIL) is frequently characterised by an initial peak in BV-associated taxa, such as *Gardnerella* and *Prevotella*, alongside early *Lactobacillus* reduction. However, as disease progresses to high-grade lesions (CIN2/3/HSIL), these specific taxa enrichment often transitions into a broader ecological collapse. High-grade squamous intraepithelial lesions (HSILs) are most consistently defined by profound *Lactobacillus* depletion and the establishment of a highly diverse, polymicrobial community (CST IV) rather than the overgrowth of a single specific pathogen. These stage-specific taxa findings are detailed comprehensively for each included cohort in Table S4.

Beyond specific taxonomic changes, most stage-specific comparisons relied on broad ecological metrics like diversity and CSTs, resulting in a heterogeneous overall picture. Several studies reported a clear stepwise gradient: alpha diversity rose progressively from normal through HPV, low-grade squamous intraepithelial lesion (LSIL), HSIL and cancer in Chen et al. (2020) [15]; diversity increased stepwise from non-squamous intraepithelial lesion to CIN3 in Liu, Wang, et al. (2023) [29] (*p* < 0.001); and diversity indices rose significantly across healthy control to HPV to CIN in Zhang et al. (2018) [38] and Zhang et al. (2022) [39]. Guo et al. (2022) [20] found that SIL severity, rather than HPV status, was the stronger driver of compositional separation, with the largest shift specifically between LSIL and HSIL.

By contrast, several studies found no additional significant shift once low-grade dysplasia was already present: Dai et al. (2025) [17] and Lee et al. (2020) [26] found no significant difference in *Lactobacillus* depletion, diversity, or CST distribution between CIN1 and CIN2+; Zeng et al. (2023) [45] found no significant difference in richness between CIN1− and CIN2+; and So et al. (2020) [35] found a significant HPV+ vs. HPV− difference (*p* = 0.002) but no significant association with CIN3 status overall. A further subset suggested dysbiosis becomes significant only once high-grade disease is present: Xu et al. (2023) [36] found HPV-positive women without CIN did not differ significantly from HPV-negative controls (*p* = 0.103), whereas CIN2+ did (*p* = 0.026), and Fan et al. (2024) [19] found major dysbiosis was apparent only at the cervical cancer stage. Conversely, a minority of studies suggested dysbiosis is already near-maximal at HPV infection itself: Pu et al. (2025) [33] found an anaerobe-dominant cluster present in 100% of HPV-positive women without neoplasia, decreasing slightly to 88% in CIN, and Liu, Li, et al. (2023) [28] found BV/HPV-associated taxa peaked at CIN1 before declining at CIN2+ as HPV status became the dominant compositional driver. Together, these findings indicate that cervicovaginal dysbiosis is consistently associated with HPV infection and CIN, but the relationship is not uniformly progressive across cohorts. Notably, these studies varied considerably in their control-group definitions and statistical power.

Few other genera showed inconsistent findings across studies: *Enterococcus* was reported as increased in four studies [13,34,36,37] and decreased in two [20,42]. *Bifidobacterium* was reported as decreased in two studies [14,39] and increased in one [18].

Among the 20 studies reporting CST data, Chen et al. (2020) [15] reported CST IV rising from 41% in HPV-positive women without CIN to 89% in cervical cancer, dipping to 35% at LSIL; groups in this study were defined by histology rather than by HPV status within each lesion grade, so the HPV-positivity rate specifically within the LSIL group is not reported. Mitra et al. (2015) [30] observed CST IV rising stepwise from 10% in controls to 21% in LSIL, 27% in HSIL, and 40% in invasive cancer, although this was statistically non-significant, which the authors attributed to modest sample size (the cancer subgroup comprised only 5 women). As mentioned above, Pu et al. (2025) [33] similarly found anaerobe-dominant cluster absent (0%) in HPV-negative controls becoming near-universal at the HPV-infection stage before decreasing slightly in CIN. Norenhag et al. (2024) [32] and Jung et al. (2025) [23] found CST IV prevalent in dysplasia, increasing with lesion severity, while Yang et al. (2025) [37] reported CST IV in 69.6% of cases, with CST IV-B linked to high-grade CIN. Complete data can be found in Table S4.

While most included studies did not stratify microbiome findings by patient race or ethnicity, Tossas et al. (2023) [43] demonstrated that race significantly modifies the relationship between the vaginal microbiome and cervical disease. In a cohort of 3050 women, the authors found that a *Lactobacillus*-dominated (optimal) microbiome was protective against CIN3 for Non-Latina White women. However, this optimal microbiome did not confer the same protection for Non-Latina Black women, who maintained a significantly higher risk of CIN3 even when harbouring an optimal microbial profile. These findings highlight that the protective mechanisms of the cervicovaginal microbiome may not be uniformly applicable across all ethnic groups.

### 3.4. Alpha and Beta Diversity Associated with HPV and CIN

**Figure 3:**
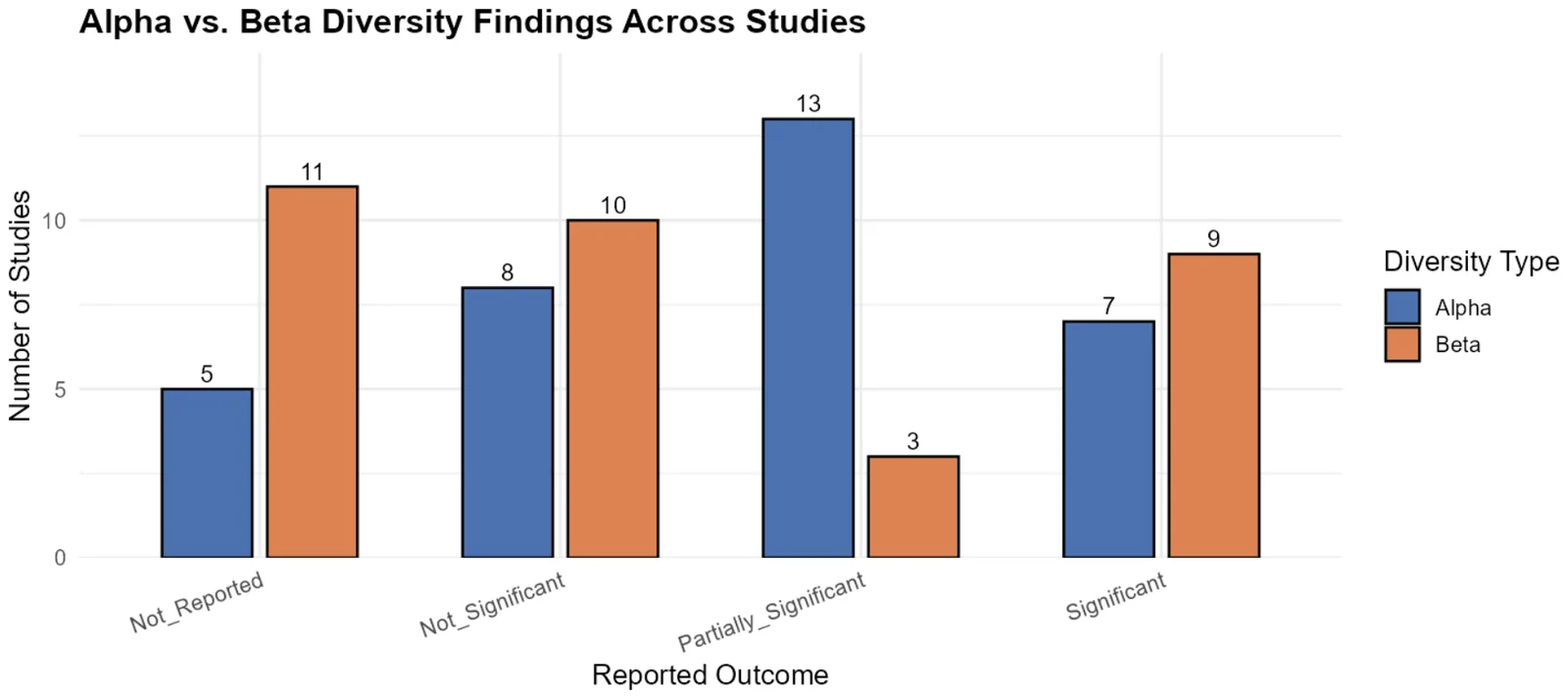
Bar chart summarising the number of studies reporting each outcome category (Not Reported, Not Significant, Partially Significant, Significant) for alpha diversity (blue) and beta diversity (orange).

Alpha diversity typically showed only partial significance (39.4%, 13 of 33 studies) [14,18,19,20,23,27,33,35,36,37,41,44,45], whereas beta diversity yielded more mixed findings, with 27.3% of studies reporting significant differences (9 of 33 studies) [14,19,20,21,25,28,32,41,43] and 30.3% reporting non-significant differences (10 of 33 studies) [16,17,26,29,33,36,38,40,42,45] (Figure 3; Figures S1 and S3 respectively). To better illustrate these variations, the exact reported numerical *p*-values and their distribution across both alpha and beta diversity metrics are compiled in Figure S1.

When examining specific quantitative indices, Chao et al. (2021) observed significantly higher Shannon diversity in HSIL (mean 2.85 ± 0.45) compared to HPV-positive women without neoplasia (mean 2.30 ± 0.50) (*p* = 0.0065) [14]. Similarly, Huang et al. (2018) reported significant increases in species richness, with mean Chao1 indices elevating from baseline controls (74.85) to high-risk HPV groups (ranging from 121.89 in HPV58 to 147.17 in HPV16) (*p* < 0.05) [21]. Additional cohorts confirmed these robust alpha diversity changes; for example, Norenhag et al. (2024) reported significant increases in the Shannon index (*p* < 0.002) and species richness (*p* < 0.001) in dysplasia versus healthy controls [32], and Lin et al. (2022) noted elevated Shannon (F = 6.14, *p* = 0.023) and Simpson indices (F = 9.49, *p* = 0.006) in HPV-positive women [27].

Conversely, beta diversity yielded highly variable compositional shifts. For instance, Lee et al. (2020) demonstrated highly significant community state type (CST) clustering separation associated with disease severity (Adonis *p* < 0.001) [26], and Norenhag et al. (2024) reported significant compositional separation between healthy controls and dysplasia (PERMANOVA *p* < 0.001, R^2^ = 0.022) [32]. In contrast, Dai et al. (2025) found only slight visual separation on PCoA with no significant shift between CIN1 and CIN2+ stages [17].

Concordance between the two metrics also varied at the study level: some studies (e.g., Huang et al. 2018, Norenhag et al. 2024, Liu, Li, et al. 2023) [21,28,32] showed significant differences in both, while others showed discordant patterns. Chen et al. (2020) reported significant alpha differences but only partial beta separation [15], while Cheng et al. (2020) and Liu, Wang, et al. (2023) reported significant alpha differences without significant beta separation [16,29]. Chao et al. (2020) showed the opposite, with non-significant Shannon diversity but significant ANOSIM separation [13]. This suggests that overall taxonomic richness and community composition do not always shift in parallel, highlighting that beta-diversity measures may capture important compositional changes that are not reflected by summary alpha-diversity indices.

### 3.5. Microbial Mechanisms Linking Dysbiosis to HPV Persistence and CIN Progression

It is important to distinguish that while microbial compositional shifts were directly observed within the included cohorts, the biological mechanisms driving progression are predominantly plausible explanations synthesised from external in vitro literature rather than tested directly in vivo. Exceptions include direct in vivo metabolomic profiling by Ilhan et al. (2019) demonstrating altered hippurate metabolism [22] and Pu et al. (2025) showing glycerophospholipid dysregulation [33]. Most studies, however, rely on indirect inferences to deduce these underlying mechanisms.

Dysbiosis may contribute to HPV persistence and CIN progression through two primary pathways (see Table 2). First, enzymatic degradation is thought to dismantle the cervicovaginal mucosal barrier, facilitated by sialidase-producing *Gardnerella vaginalis* and *Prevotella* spp. [15,18,27,28], alongside proteolytic *Porphyromonas* spp. and *Streptococcus* spp. [24,37]. Second, dysbiotic taxa orchestrate immune modulation, facilitating viral evasion via targeted immunosuppression (e.g., *Gardnerella*-mediated IL-17 reduction [44]) or by sustaining chronic inflammation associated with *Streptococcus* spp. and *Corynebacterium* spp. [24]. *Lactobacillus iners* was a notable exception among otherwise protective lactobacilli, producing inerolysin and independently associated with high-risk HPV persistence [42,45]. At the community level, the CST IV profile reflects these mechanisms with altered lactic acid ratios, elevated MMP-8, and EMMPRIN [29].

In addition to enzymatic and immune modulation, alterations in the vaginal pH environment were noted as a functional consequence of dysbiosis in four studies (Table S5). Ilhan et al. (2019) [22] found a higher pH in cervical cancer (6.6) than in controls (4.9). Pu et al. (2025) [33] reported elevated pH in HPV-positive (6.95 ± 0.77) and CIN groups (7.02 ± 1.18) compared to healthy controls (5.99 ± 0.92, *p* < 0.001). Morales et al. (2022) [31] noted 52% had abnormal pH (>4.5). Dou et al. (2024) [18] saw no significant pH difference between HPV-positive and HPV-negative groups.

## 4. Discussion

### 4.1. Summary of Findings

**Figure 4:**
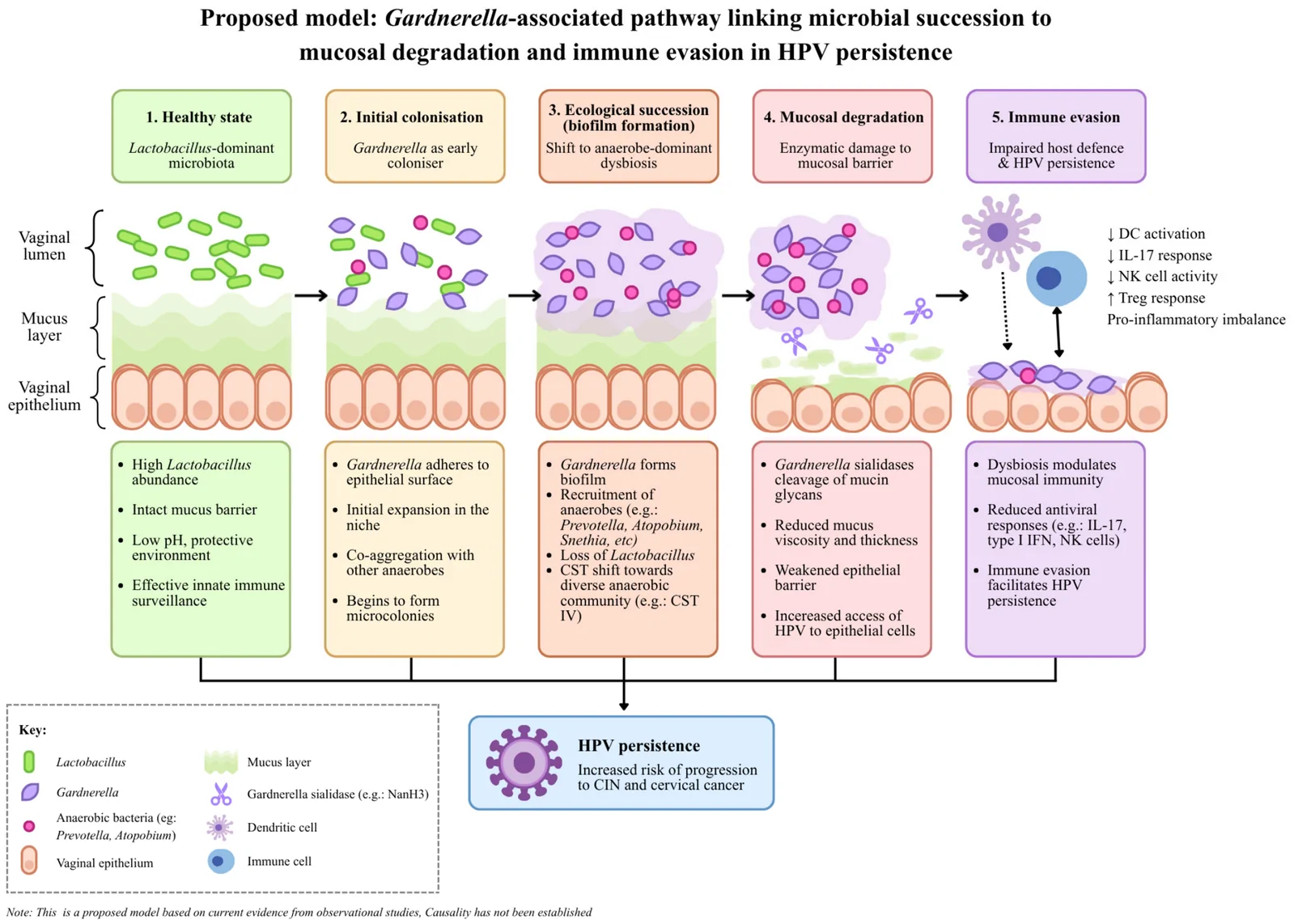
Proposed model of *Gardnerella*-associated mucosal degradation and HPV persistence. Based on the mechanistic data from the included studies, *Gardnerella* acts as an early coloniser and uses sialidases to break down the protective mucin barrier [15,18,27]. This enzymatic breakdown promotes epithelial adhesion and supports ecological succession, leading to a diverse, anaerobic biofilm community (e.g., CST IV) [18,32]. This dysbiotic shift triggers an immunosuppressive microenvironment, marked by decreased antiviral responses such as IL-17 and NK cells, along with heightened regulatory T-cell activity. This environment ultimately supports HPV persistence and raises the risk of neoplastic progression [44]. Abbreviations: CIN, cervical intraepithelial neoplasia; CST, community state type; DC, dendritic cell; HPV, human papillomavirus; IFN, interferon; IL-17, interleukin-17; NK, natural killer; Treg, regulatory T cell.

Across nearly all microbial phyla, a consistent pattern emerged: HPV-positive and CIN samples were characterised by a depletion of protective *Lactobacillus* and a broad enrichment of diverse anaerobic and facultative taxa, including *Gardnerella*, *Prevotella* and *Sneathia*. Two earlier meta-analyses, Brusselaers et al. and Liang et al., quantified the epidemiological association between vaginal dysbiosis and HPV/cervical cancer risk but did not identify which taxa contribute to this association [46,47]. By mapping functional evidence such as enzymatic barrier degradation and immune modulation, this review establishes a plausible biological pathway linking the transition to a high-diversity, anaerobe-dominant microbiome to viral persistence and neoplastic progression (Figure 4).

### 4.2. Microbial Taxa Changes Across Disease Stages

Our findings showed broadly consistent directional changes despite considerable heterogeneity in sequencing platforms, target regions, geographical settings, and comparison-group definitions across the included studies. The consistency of these findings across such methodologically diverse studies makes them unlikely to be attributed to any single laboratory pipeline or population, instead pointing to a genuinely conserved biological relationship between anaerobic dysbiosis and HPV/CIN status.

This taxonomic profile reflects Ravel et al.’s CST framework, in which the loss of *Lactobacillus* dominance and a shift toward high-diversity CST IV increase susceptibility to genital pathogens [48], indicating that HPV- and CIN-associated dysbiosis is better seen as a concurrent *Lactobacillus* depletion and diverse anaerobic enrichment rather than a single-genus issue. *L. iners* did not follow the broader genus-level protective pattern, indicating that genus-level classification alone may not reflect the biological relevance of *Lactobacillus* species in this context.

This pattern is also consistent with an ecological succession model rather than independent replacement of each taxon. *Gardnerella* is identified in biofilm research as an initial coloniser that establishes a biofilm structure, enabling secondary anaerobes such as *Prevotella* spp. and *Fannyhessea vaginae* to adhere subsequently [49]. However, summary study counts, such as *Lactobacillus* depletion reported in the 24 of 27 cross-sectional reports, must be interpreted with care. While these counts are descriptively helpful to highlight consistent taxonomic shifts, they do not show the effect size or statistical reliability of an association with any given cohort. The included studies differ substantially in their target populations, definitions of comparison groups, and sequencing methods. Furthermore, some reports (e.g., Chao et al. 2020 and 2021 [13,14], or Cheng et al. 2020 and Norenhag et al. 2024 [16,32]) are likely to contain overlapping participants drawn from the same underlying cohorts. These methodological variations can skew individual taxon counts. Consequently, the primary findings represent general ecological patterns rather than precise quantitative risk metrics.

Framing it this way suggests that the co-occurrence of *Gardnerella*, *Prevotella*, and *Sneathia* across studies indicates an organised community, not just separate disease factors. This distinction determines if targeting a single taxon, like *Gardnerella*, can disrupt the entire dysbiotic community or if secondary colonisers persist. Alongside well-established dysbiotic genera, *Dialister* emerged as a highly consistent biomarker of disease across multiple independent cohorts. Five studies found *Dialister* more prevalent in patients with persistent HPV infection or progressive CIN compared to baseline or healthy controls [18,27,28,30,34]. No opposing trends for this genus were observed in the studies. As an anaerobic bacterium associated with diverse CST IV microbiomes, *Dialister* shows a consistent increase across these populations, highlighting its role as a core component of the pro-inflammatory microbial structure that promotes disease progression.

While most reported taxa demonstrated directional consensus, *Enterococcus* exhibited conflicting patterns across studies, being reported as both increased [33,35] and decreased [19,41] in disease states. Because *Enterococcus* is typically a low-abundance taxon in the vaginal microbiome, its accurate detection is highly sensitive to the specific hypervariable region targeted during amplification. A comparison of these specific studies reveals extreme methodological heterogeneity, with each utilising a different 16S rRNA target region: V3–V4 [19], a 5-region panel [33], V4 [35] and V1–V2 [41]. Furthermore, the studies utilised different sequencing platforms (Illumina NovaSeq 6000 vs. MiSeq) and study designs (cross-sectional vs. longitudinal). This extreme heterogeneity indicates that primer bias and methodological variances, rather than true biological differences, likely drive these conflicting results.

### 4.3. Alpha and Beta Diversity Patterns

Alpha and beta diversity findings across the included studies were less consistent than the taxonomic shifts described above. At the individual study level, this divergence was evident: Chen et al. (2020) reported significant alpha diversity differences but only partial beta diversity separation [15], whereas Tossas et al. (2023) and Zhang et al. (2022) showed the opposite pattern, with non-significant or unreported alpha diversity but significant beta diversity separation [39,43]. Much of this difference likely reflects the specific diversity metrics discussed in the literature. Since vaginal dysbiosis involves the loss of *Lactobacillus* dominance, metrics that capture community evenness such as the Shannon index are more sensitive to this shift than richness estimators like Chao1, which simply count taxa. Similarly, abundance-weighted beta diversity metrics, such as the Bray–Curtis metrics, are highly responsive to the replacement of a dominant genus with diverse anaerobes. This explains why beta diversity often reveals significant community differences, even when overall richness remains stable. This discordance between alpha and beta diversity was less evident in Wu et al.’s (2022) synthesis, which reported significantly increased diversity across HPV-related LSIL and HSIL [5]. Such differences may stem from variations in disease-staging granularity and the specific diversity metrics applied across the two syntheses. Ultimately, the clinical relevance of the CST framework depends on whether these compositional changes produce noticeable functional effects on the host mucosa, a dynamic discussed below.

### 4.4. Functional and Mechanistic Links to Pathogenesis

The functional and mechanistic data support the biological plausibility of the listed taxonomic links. A common mechanism involves the enzymatic breakdown of the cervicovaginal mucosal barrier, in which *Gardnerella vaginalis* and *Prevotella* spp. produce sialidase, while *Porphyromonas* spp. and *Streptococcus* spp. contribute proteolytic and metallopeptidase activities, respectively [15,18,24,26,27,28,37]. *Gardnerella* sialidases, notably those encoded by the nanH3 gene, cleave mucin oligosaccharides, reducing mucus viscosity and weakening the epithelial barrier. This potentially facilitates HPV access into basal keratinocytes and exposes protective mucosal IgA to proteolytic degradation [50,51]. Consistent with this, women with persistent HPV16 infections exhibit significantly higher baseline nanH3 gene loads in their cervicovaginal fluid compared to women who clear the virus through immune mechanisms [52]. A second theme was immune modulation, which took two seemingly different yet possibly converging paths: immunosuppression, demonstrated by *Gardnerella vaginalis* reducing IL-17 levels and *Fusobacterium nucleatum* inhibiting natural killer cell cytotoxicity [18,27,44,45], and chronic pro-inflammatory activation, driven by *Streptococcus* and *Corynebacterium* spp. [24,26,37]. At a molecular level, *F. nucleatum* utilises the Fap2 surface protein to directly bind and activate TIGIT, an inhibitory receptor present on T cells and natural killer (NK) cells, thereby shielding the microenvironment from immune-mediated clearance [53].

That both immunosuppressive taxa and chronic pro-inflammatory taxa are associated with disease progression may seem contradictory, but is consistent with a model in which sustained, dysregulated inflammation itself impairs effective antiviral immunity, meaning the net effect of either route converges on the same functional immune failure. Persistent pro-inflammatory taxa weaken immunity and promote viral-driven cancer development. Ongoing mucosal inflammation leads to elevated reactive oxygen species (ROS) and oxidative stress, causing DNA double-strand breaks, genomic instability, and insertion sites that facilitate HPV integration into the host genome, essential for progression from low-grade to high-grade CIN [54].

*L. iners* also emerged as an important exception within an otherwise protective genus. Unlike highly protective species such as *L. crispatus*, *L. iners* produces only L-lactic acid rather than D-lactic acid, and secretes inerolysin, a cholesterol-dependent cytolysin that directly induces host cell membrane damage. A high vaginal ratio of L-lactate to D-lactate elevates extracellular matrix metalloproteinase inducer (EMMPRIN), which subsequently activates matrix metalloproteinase-8 (MMP-8), degrading epithelial tight junctions. Coupled with a lack of protective bacteriocin-synthesis genes, this metabolic profile explains why *L. iners* is independently associated with the failure to clear high-risk HPV, shifting the microenvironment toward dysbiosis [42,45]. This offers a mechanistic explanation for genus-level heterogeneity and highlights the importance of species-level analysis when interpreting *Lactobacillus* data. At the community level, the typical CST IV profile combined an altered L-to-D-lactic acid ratio with elevated MMP-8 and EMMPRIN levels, mirroring the enzymatic activity and barrier-disrupting mechanisms previously identified in individual taxa [29,35]. Beyond altered lactic acid ratios, the shift to a diverse CST IV network results in a unique metabolomic profile characterised by biogenic amines, including putrescine and cadaverine, and short-chain fatty acids, including succinate and acetate [55]. These anaerobic metabolites increase vaginal pH, weakening the acidic barrier that limits pathogens. They also inhibit monocyte chemotaxis and dendritic cell maturation, increasing immunosuppression [56].

Consistent with this enzymatic and immune-mediated model, alterations in vaginal pH, reported in four included studies, offer an additional, low-cost functional correlate of dysbiosis. Elevated pH in HPV-positive and CIN samples relative to controls parallels the loss of lactic-acid-producing *Lactobacillus*, although this was not universal: Dou et al. found no significant pH difference between HPV-positive and HPV-negative groups [18], underscoring that pH alone cannot substitute for taxonomic assessment.

It is also important to recognise that the mechanistic evidence in Table 2 robustly supports the earlier taxonomic findings through a combination of internal convergence and external triangulation. Specifically, the functional findings regarding sialidase activity and targeted immune modulation demonstrate strong within-study convergence, as multiple included primary cohorts independently report corresponding taxonomic and functional shifts (e.g., *Gardnerella* enrichment paired with mucosal degradation or altered cytokine profiles) [15,18,24,26,27,28,32,37,44,45]. Furthermore, these internal cohort findings are externally triangulated by an independent body of in vitro and biochemical literature. This external literature validates the broader mechanisms facilitating disease progression, including ROS-mediated DNA damage [54] and overarching metabolomic dysregulation [55,56]. By explicitly synthesising both within-study functional correlates and independent mechanistic literature, this approach offers a more rigorous biological foundation for pathogenesis than previous reviews that typically focused on taxonomic composition alone.

### 4.5. Longitudinal Evidence and Causal Inference

Due to the cross-sectional design of most studies, a critical question of directionality remains: does vaginal dysbiosis facilitate HPV persistence, or does the viral infection itself trigger dysbiosis? The six longitudinal studies in this review [40,41,42,43,44,45] are especially significant because only repeated sampling of the same women over time can establish a temporal sequence.

Two studies support the idea that dysbiosis may come before disease progression, not just occur alongside it. Usyk et al. studied women with new high-risk HPV infections across two visits. They identified *Gardnerella* dominance at the initial visit as the key baseline indicator for future progression to CIN2+. Mediation analysis revealed that this association was directly mediated by an increase in microbial diversity at the second visit [44]. Thus, *Gardnerella* dominance statistically accounted for the transition to a highly diverse state, which in turn preceded clinical progression. Similarly, Mitra et al. (2020) repeatedly sampled women with confirmed, untreated CIN2 over 24 months and found that a *Lactobacillus*-dominant microbiome at baseline, before any regression had occurred, predicted subsequent regression. Conversely, baseline *Lactobacillus* depletion along with *Megasphaera*, *Prevotella timonensis*, and *Gardnerella vaginalis* was associated with persistence [41]. In both studies, the microbiome was measured prior to the clinical outcomes it predicted, ensuring the proper temporal order that many cross-sectional studies fail to establish.

The remaining four longitudinal studies offer further, if more modest, support for a temporal relationship between microbiome composition and disease trajectory. Berggrund et al. (2020) [40] found that a dysbiotic cluster at baseline was only marginally associated with HPV persistence and did not remain significant after multiple testing correction. However, the instability of the microbial profile over time, rather than a fixed dysbiotic state, was associated with an increased likelihood of transitioning to persistent infection. Shi (2022) [42] found that high baseline relative abundance of *L. iners* predicted subsequent non-clearance of HPV, particularly within the LSIL subgroup (adjusted OR 3.94). Tossas et al. (2023) [43] observed a significant increasing trend in vaginal microbiome diversity across the trajectory from HPV clearance to persistence to progression (*p* = 0.024), though this relationship was modified by race. Similarly, Zeng et al. (2023) [45] found that women with persistent HPV reported significantly higher microbial richness at follow-up (*p* = 0.046) than those who cleared the virus. Together, these four studies reinforce an association between a less stable, more diverse microbiome and unfavourable HPV/CIN trajectories, though none demonstrates temporal precedence as clearly as Usyk et al. [44] and Mitra et al. (2020) [41].

These findings should not be interpreted as evidence of causation. Unmeasured confounders, such as sexual behaviour or immune status, might independently influence both microbiome composition and disease progression. Neither study experimentally manipulated the microbiome to test if changes affect outcomes. Nevertheless, when considering the graded relationship shown in Chen et al.’s cross-sectional data, where CST IV prevalence increased stepwise from healthy controls to HPV-positive women, and ultimately to cervical cancer patients [15], the sequence in these cohorts strengthens the evidence for a consistent link rather than mere correlation, surpassing what cross-sectional data alone can show.

### 4.6. Strengths and Limitations

A major strength of this review is its departure from purely descriptive taxonomy. While previous syntheses have established epidemiological links between dysbiosis and HPV, this review uniquely bridges structural data (taxonomic shifts and diversity metrics) with functional evidence (enzymatic barrier degradation and immune modulation). By mapping these specific biological mechanisms, this review establishes a cohesive, biologically plausible framework explaining exactly how a high-diversity, anaerobe-dominant community may facilitate viral persistence and neoplastic progression. Moreover, the thorough methodology of this review enabled the detection of often-overlooked taxonomic agreements, such as the repeated enrichment of *Dialister*, which was not identified as a key marker in earlier analyses. It comprehensively examined the full range of reported vaginal microbial taxa, extending beyond earlier reviews that focused exclusively on *Lactobacillus* [4], restricted analysis to a single population [6], or explicitly excluded functional profiling [5].

Several limitations should also be acknowledged. The significant heterogeneity across sequencing platforms, bioinformatics pipelines, and study populations prevented meta-analysis, so this review relied on narrative synthesis, which precludes the calculation of quantitative pooled estimates. This narrative synthesis itself relies substantially on vote-counting the frequency of directional findings across studies (e.g., *Lactobacillus* reported as depleted in 24 of 27 reports, 89%); this approach does not weight studies by sample size, effect size, or methodological quality, and could overstate the strength of an association if a small number of large, high-quality studies were numerically outweighed by several smaller, lower-quality ones reporting the same direction of effect. The exclusion of grey literature and conference abstracts during screening could have caused publication bias, since studies showing null or non-significant links between vaginal microbiome and HPV/CIN outcomes are less often published in peer-reviewed journals.

Secondly, the evidence base was geographically biased, with more than half of the studies conducted in China and few from Africa or other regions with high cervical cancer burden, which restricts generalizability. The evidence base is also not fully independent at the study level: Chao et al. (2020) [13] and Chao et al. (2021) [14] share the same ethics approval and trial registration (NCT03548740), indicating likely overlap in the underlying participant cohort; this should be considered when interpreting the consistency of directional findings across included reports.

The limited geographical and population representation of the available evidence also restricts understanding of how host–microbiome interactions may vary across different populations at risk of cervical cancer. Additionally, the dominance of cross-sectional studies significantly limits the ability to establish causality, creating an ongoing dilemma of bidirectionality. It remains unclear whether the decrease in *Lactobacillus* leads to HPV persistence or whether changes in the microenvironment of a neoplastic lesion, such as local inflammation, metabolic shifts, or alterations in mucosal pH, favour opportunistic anaerobic bacteria. Nonetheless, the six longitudinal studies included in this review provide preliminary insights into the potential directions of these relationships.

Furthermore, the heavy reliance on 16S rRNA amplicon sequencing in these studies presents a significant limitation in evaluating microbial functions. Since 16S sequencing provides only taxonomic insights and does not directly reveal functional activity, identifying virulence factors such as *Gardnerella* sialidases or *Fusobacterium* Fap2 proteins primarily relies on detecting their associated genera. Without integrating more comprehensive methods such as shotgun metagenomics or meta-transcriptomics, the findings are largely correlational and cannot confirm whether these pathogenic genes are actively expressed in the cervical microenvironment. Furthermore, although a shift to a high-diversity, anaerobe-dominant state (CST IV) is often linked to HPV persistence and CIN progression, most studies did not report bacterial vaginosis (BV) diagnoses based on standard clinical metrics, such as the Nugent score or Amsel criteria. As a result, it remains unclear how many women have clinically diagnosed BV compared to those with dysbiosis identified through molecular methods, including asymptomatic cases, across these cohorts. This highlights a significant gap between microbiome sequencing outcomes and conventional clinical diagnostics.

### 4.7. Clinical Implications and Future Directions

Clinically, the consistent observation of *Lactobacillus* depletion alongside enrichment of *Gardnerella*, *Prevotella,* and other dysbiosis-associated taxa suggests that vaginal microbiome profiling could serve as a valuable prognostic tool for identifying women at increased risk of HPV persistence or CIN progression. These findings highlight the therapeutic potential for microbiome-targeted interventions, such as probiotics or live biotherapeutics, to restore the prevalence of beneficial, non-iners *Lactobacillus* species [57], although current evidence is mainly observational and insufficient to support clinical use. To bridge the gap between observation and clinical application, future research should transition from cross-sectional to multi-timepoint longitudinal studies to monitor microbiome changes and clinical outcomes. To directly link taxonomy to biological function, the field must move beyond 16S rRNA sequencing by integrating shotgun metagenomics with targeted anaerobic bacterial culturing. This approach enables detailed functional gene mapping and isolation of colonies for mechanistic studies and therapy testing.

## 5. Conclusions

This review establishes a consistent, biologically coherent signature of vaginal dysbiosis, characterised by *Lactobacillus* depletion and anaerobic enrichment, that accompanies HPV persistence and CIN progression. Linking these compositional shifts to convergent enzymatic (sialidase-mediated barrier degradation) and immunomodulatory mechanisms and identifying *Lactobacillus iners* as a species-level exception to genus-level protection, this review moves beyond descriptive taxonomy toward a mechanistic account of how the cervicovaginal microenvironment may shape cervical carcinogenesis. Importantly, the six included longitudinal studies provide early evidence of temporal precedence, demonstrating sequential associations between baseline dysbiosis and disease progression, but causal inference remains limited by the cross-sectional design of most of the included work. Validating these relationships and translating them into microbiome-based risk stratification or therapeutic strategies will require prospective, multi-omics research that combines functional insights with taxonomy. Such studies should include more diverse populations ethnically and geographically, especially in Africa and Southeast Asia, regions with a high cervical cancer burden but limited representation in existing microbiome research.

## Figures and Tables

**Table 1:** General characteristics of included studies.

References	Country	Study Design	Comparison Groups ^1^	Mean Age (Range), Years ^2^	HPV Detection Method + Genotypes ^1,3^	Sequencing Platform/Target Region ^4^
Chao et al. (2020) [13]	China	Cross-sectional	Persistent hrHPV (*n* = 59)	42	Cobas 4800 (HR types)	16S rDNA (V4), Illumina HiSeq2500
			Incident hrHPV (*n* = 139)	41		
			HPV− (*n* = 131)	42		
Chao et al. (2021) [14]	China	Cross-sectional	HSIL (*n* = 83)	38	Cobas 4800 (HR types)	16S rDNA (V4), Illumina HiSeq2500
			HPV+ no neoplasia (*n* = 86)	39		
			HPV− (*n* = 103)	39		
Chen et al. (2020) [15]	China	Cross-sectional	Healthy control (*n* = 68)	43 (25–69)	hrHPV (method not reported)	16S rRNA (V3–V4), Illumina MiSeq
			HPV (*n* = 78)	48 (25–69)		
			LSIL (*n* = 51)	46 (25–69)		
			HSIL (*n* = 23)	44 (25–69)		
			Cancer (*n* = 9)	56 (25–69)		
Cheng et al. (2020) [16]	Sweden	Cross-sectional	HPV-positive (*n* = 144) vs. HPV-negative (*n* = 113) Oncogenic HPV (*n* = 81) vs. Non-oncogenic HPV (*n* = 10) vs. Mixed (*n* = 42) vs. Uninfected (*n* = 113) Single infection (*n* = 51) vs. Multiple infections (*n* = 93) Youth clinic (*n* = 169) vs. Cervical screening (*n* = 88)	14–29	Multiplex bead-based Luminex MAGPIX assay	16S rRNA (V3–V4), Illumina MiSeq
Dai et al. (2025) [17]	China	Cross-sectional	HPV+ CIN1 *n* = 23	34 (27–48)	Cobas 4800	16S rRNA (V4–V5), Illumina NovaSeq
			HPV+ CIN2+ *n* = 20	31 (23–46)		
Dou et al. (2024) [18]	China	Cross-sectional	HPV+ (*n* = 70)	<35 years (*n* = 86) ≥35 years (*n* = 50)	PCR + hybridization	16S rRNA (V3–V4), Illumina NovaSeq 6000
			HPV− (*n* = 136)	<35 years (*n* = 43) ≥35 years (*n* = 27)		
Fan et al. (2024) [19]	China	Cross-sectional	Healthy control (*n* = 27)	≤50 years (*n* = 24) >50 years (*n* = 3)	PCR reverse-hybridisation (28 types)	16S rRNA (V3–V4), Illumina NovaSeq 6000
			hrHPV (*n* = 40)	≤50 years (*n* = 32) >50 years (*n* = 8)		
			CIN2/3 (*n* = 40)	≤50 years (*n* = 31) >50 years (*n* = 9)		
			Cervical Cancer (*n* = 18)	≤50 years (*n* = 10) >50 years (*n* = 8)		
Guo et al. (2022) [20]	China	Cross-sectional	Healthy control (HPV−) (*n* = 30)	39 (29–49)	Cobas 4800 (14 HR types)	16S rDNA (V4–V5), Illumina NovaSeq
Huang et al. (2018) [21]	China	Cross-sectional	HPV+ No SIL (*n* = 40)	38 (22–50)	HPV GenoArray (21 types)	16S rRNA (V4–V5), Illumina MiSeq
Ilhan et al. (2019) [22]	USA	Cross-sectional	HPV+ LSIL (*n* = 28)	34 (21–47)	Linear Array (37 types)	16S rRNA (V4) & LC-MS Metabolomics
Jung et al. (2025) [23]	South Korea	Cross-sectional	HPV+ HSIL (*n* = 51)	32 (19–47)	qPCR (Anyplex) + shotgun	Shotgun metagenomics, DNBSEQ-G400
			Healthy control (LSIL, HPV 16/18) (*n* = 9)	41		
			HSIL, other hrHPV (*n* = 12)	38		
			HSIL, HPV 16/18 (*n* = 11)	38		
			ICC, other hrHPV (*n* = 12)	56		
			ICC, HPV 16/18 (*n* = 10)	44		
Kazakova et al. (2024) [24]	Russia	Cross-sectional	HSIL (*n* = 20)	36	hrHPV+ (inclusion only)	No sequencing
			NILM (*n* = 20)	35		
Lee et al. (2013) [25]	South Korea	Cross-sectional	Healthy control (HPV−, CIN−) (*n* = 26) HPV+, CIN− (*n* = 19) HPV−, CIN+ (*n* = 1) HPV+, CIN+ (*n* = 4)	31–73 *	MY09/11 + GP5+/6+	16S (V2–V3), 454 pyrosequencing
Lee et al. (2020) [26]	South Korea	Cross-sectional	CIN 1− (healthy control to CIN1) (*n* = 24)	49	Anyplex II HPV28	16S (V3), Ion Torrent PGM
			CIN 2+ (CIN2 to cervical cancer) (*n* = 42)	42		
Lin et al. (2022) [27]	China	Cross-sectional	HPV+ (*n* = 138) HPV− (*n* = 310)	Not reported	PCR-RDB (18 HR/5 LR)	16S rRNA (V4), Illumina MiSeq
			CIN1+ (*n* = 51) (CIN I, II, III or cervical cancer)	CIN1+: 40		
			Healthy control (*n* = 379)	Normal: 37		
			Control group (NILM) (*n* = 126) Case (≥CIN I) (*n* = 51)	Not reported		
			HPV+ (*n* = 23) HPV− (*n* = 5)	Not reported (*p* = 0.666)		
Liu, Li, et al. (2023) [28]	China	Cross-sectional	Non-BV HPV− (*n* = 414) Non-BV HPV+ (*n* = 108) BV HPV− (*n* = 330) CIN1 (*n* = 38) CIN2+ (*n* = 39)	45 (21–64) *	BOHUI 14 hrHPV assay	16S rRNA (V4), Illumina MiniSeq
Liu, Wang, et al. (2023) [29]	China	Cross-sectional	NSIL (*n* = 279)	35	hrHPV+ (inclusion only)	16S rRNA (V4), Illumina NovaSeq 6000
			CIN1 (*n* = 233)	34		
			CIN2 (*n* = 147)	35		
			CIN3 (*n* = 33) (all hrHPV+)	36		
Mitra et al. (2015) [30]	United Kingdom	Cross-sectional	Healthy control (*n* = 20)	31 (24–43)	Abbott RealTime (HR types)	16S rRNA (V1–V2), Illumina MiSeq
			LSIL (*n* = 52)	32 (23–45)		
			HSIL (*n* = 92)	30 (25–43)		
			ICC (*n* = 5)	34 (27–41)		
Morales et al. (2022) [31]	Brazil	Cross-sectional	hrHPV+ (*n* = 86) hrHPV− (*n* = 460) CST I (*n* = 166) CST II (*n* = 27) CST III (*n* = 204) CST IV (*n* = 143) CST V (*n* = 6)	34 (18–51) *	Linear Array (36 types)	16S rRNA (V3–V4), Illumina MiSeq
Norenhag et al. (2024) [32]	Sweden	Cross-sectional	Healthy controls (*n* = 177)	35 (23–68)	HPVir real-time PCR; 27-plex Luminex bead-based assay; HPViewer	Shotgun Metagenomics, DNBSEQ-T7 (100 bp paired-end)
			Dysplasia (*n* = 177)	35 (21–70)		
			LSIL (*n* = 81)	37 (21–70)		
			HSIL/cancer (*n* = 96)	34 (22–62)		
Pu et al. (2025) [33]	China	Cross-sectional	Healthy control (HPV−) (*n* = 27)	40	HR/medium-low typing	16S rRNA 5-region, Illumina NovaSeq 6000
			Healthy control (HPV+) (*n* = 24)	36		
			CIN (*n* = 43) (CIN1 *n* = 9, CIN2/3 *n* = 34)	34		
Santella et al. (2022) [34]	Italy	Cross-sectional	HPV+ (*n* = 25) HPV− (*n* = 6)	Not reported	Linear Array (37 types)	16S rDNA (V3–V4–V6), Illumina MiSeq
So et al. (2020) [35]	South Korea	Cross-sectional	HPV− (*n* = 10) HPV+ (*n* = 10) HPV+ CIN1 (*n* = 10) HPV+ CIN2/3 (*n* = 10) HPV+ invasive squamous cell carcinoma (*n* = 10)	Not reported	hrHPV (method not reported)	16S rRNA (V4–V4), Illumina MiSeq
Xu et al. (2023) [36]	China	Cross-sectional	HRCD (*n* = 98) Healthy control (*n* = 58)	Not reported	Genotype NR	16S rRNA (V3), Illumina MiSeq
Yang et al. (2025) [37]	Taiwan	Cross-sectional	CIN2+ (*n* = 57) CIN1 (*n* = 25) HPV+ (no CIN) (*n* = 37) HPV− (*n* = 54)	44 *	DR.HPV kit (27 types)	16S rRNA (V3–V4), Illumina MiSeq
Zhang et al. (2018) [38]	China	Cross-sectional	Healthy control (*n* = 64) CIN1 (*n* = 62) CIN2 (*n* = 19) CIN3 (*n* = 21)	Not reported	SPF1/GP6+ PCR (17 types)	16S rRNA (V3–V4), Illumina HiSeq 2500
			Transient HPV16 (*n* = 26)	36		
			Persistent HPV16+ CIN2+ (*n* = 38)	38		
Zhang et al. (2022) [39]	China	Cross-sectional	Healthy control (*n* = 113) HPV (*n* = 159) (HPV-S *n* = 107, HPV-M *n* = 52) CIN (*n* = 84) (CIN1 *n* = 47, CIN2 *n* = 24 CIN3 *n* = 13)	20–70 *	HC2 + genotyping (20 types)	16S rRNA, Illumina NovaSeq
Berggrund et al. (2020) [40]	Sweden	Longitudinal cohort	HPV− (*n* = 32)	37	qPCR (hpVIR)	16S 7 hypervariable regions, Ion Torrent S5
Mitra et al. (2020) [41]	USA	Longitudinal cohort	Persistent HPV16+ CIN2+ (*n* = 38)	38	Linear Array (37 types)	16S rRNA (V1–V2), Illumina MiSeq
Shi (2022) [42]	China	Longitudinal cohort	HPV-cleared (*n* = 45)	Median age: 35	GenoArray (single HR subtype)	16S rRNA (V4–V5), Illumina MiSeq
			HPV-uncleared (*n* = 28)	Median: 36.5		
Tossas et al. (2023) [43]	USA	Longitudinal cohort	Non-Latina Black (*n* = 2112)	33	Self-report/EMR (genotype not reported)	16S rRNA (V1–V3)
			Non-Latina White (*n* = 908)	36		
			CIN3 (*n* = 93)	32		
			<CIN3/no CIN (*n* = 2927)	34		
Usyk et al. (2020) [44]	Costa Rica	Longitudinal cohort	Clearance (*n* = 70) Persistence (*n* = 170) Progression (CIN2+) (*n* = 33)	18–25 *	hrHPV panel (12 types)	16S rRNA (V4) & fungal ITS1, Illumina MiSeq
Zeng et al. (2023) [45]	China	Longitudinal cohort	hrHPV16/18 (*n* = 28)	Median: 37 IQR: 18	Genotype not reported	16S rDNA, Ion S5 XL
			Non-hrHPV16/18 (*n* = 62)	Median: 42 IQR: 11		
			Healthy control (*n* = 45)	Median: 36 IQR: 17		

* Age across all groups. ^1^ hrHPV, high-risk human papillomavirus; ICC, invasive cervical cancer; No SIL/NSIL, no squamous intraepithelial lesion; LSIL, low-grade squamous intraepithelial lesion; HSIL, high-grade squamous intraepithelial lesion; 16LSIL/16HSIL, HPV16-infected low-/high-grade lesion; 52LSIL/52HSIL, HPV52-infected low-/high-grade lesion; 58LSIL/58HSIL, HPV58-infected low-/high-grade lesion; HRCD, high-risk cervical disease; HPV-S, single HPV infection; HPV-M, multiple HPV infection; BV, bacterial vaginosis; CST, community state type. ^2^ IQR, interquartile range. ^3^ HR/LR, high-risk/low-risk (HPV type); HC2, Hybrid Capture 2 assay. ^4^ LC-MS, Liquid Chromatography-Mass Spectrometry; PGM, Personal Genome Machine; rDNA, ribosomal Deoxyribonucleic Acid; rRNA, ribosomal Ribonucleic Acid; ITS1, Internal Transcribed Spacer 1.

**Table 2:** Microbial enzymatic, immune, and mechanistic contributions to HPV persistence and cervical intraepithelial neoplasia progression, organised by taxon.

Microbe (Genus/Species)	Enzymatic & Metabolic Action ^1,3^	Immune & Inflammatory Action ^1,4^	Proposed Mechanism of HPV Persistence & CIN Progression (Derived from External Literature) ^3,5^	Evidence Source
Individual taxa				
*Gardnerella vaginalis*	Produces sialidase and vaginolysin/cytolysin [15,18,27]. Forms dense biofilms [18]. Identified as the main contributor to hippurate metabolism [22]. Drives peptidoglycan biosynthesis and cell-wall maturation pathways as well as L-alanine biosynthesis.	Exerts immunosuppressive effects in the cervicovaginal niche, such as reducing IL-17 [44]. Actively inhibits mucosal barrier IgA protection [18,27].	Degrades mucosal protective sialoglycans and mucins, destroying the epithelial barrier, entrapping anaerobic bacteria, facilitating HPV entry, evading host immunity, and potentially facilitating CIN progression [15,18,27]. Acts as a primary catalyst inducing elevated polymicrobial diversity, predicting CIN2+ progression [44].	Proposed based on external in vitro literature. Hippurate metabolism tested in vivo [22]
*Prevotella* spp. (e.g., *P. bivia*, *P. timonensis*)	Produces sialidase and fucosidase [28,32]. *P. bivia* produces LPS [33].	Strengthens inflammatory response and immune activation [28]. Interplays with altered lipid metabolites to sustain localised tissue inflammation [33].	Breaks down the protective mucosal layer, promoting HPV acquisition and low-grade CIN development [28,32]. Sustains cervical tissue inflammation leading to viral persistence and CIN progression [33].	Proposed based on external literature.
*Sneathia* spp. (*S. sanguinegens*, *S. vaginalis*)	Not reported at the individual species level.	Alters expression of immunosuppressive cytokines [15]. Activates proinflammatory pathways and inhibits immunocytotoxicity [20].	Adheres to malignant cervical cells and releases toxic products [37]. Impairs immune clearance and degrades mucosal barriers, facilitating persistent HPV infection and neoplastic progression [20,37].	Proposed based on external literature.
*Streptococcus* spp. (*S. anginosus*, *S. agalactiae*, *S. pyogenes*)	Possesses metallopeptidases capable of cleaving extracellular matrix proteins like fibronectin and type IV [24]. Causes active lysis of vaginal epithelial cells [26].	Activates multiple pro-inflammatory cytokines, including interleukin-17 [24]. Causes chronic inflammation [37].	Metallopeptidases facilitate bacterial penetration into tissues and dissemination [24]. Epithelial cell lysis and chronic inflammation aid viral persistence and accelerate progression from low-grade to high-grade [26,37].	Proposed based on external literature.
*Lactobacillus iners*	Produces inerolysin, a cholesterol-dependent pore-forming cytolysin [42]. Produces less-protective L-lactic acid and lacks genes for host-protective bacteriocin synthesis [42,45].	Positively correlated with pro-inflammatory cytokines in vaginal fluid [42].	Damages host cell membranes and create a pro-inflammatory microenvironment that impairs immune clearance, acting as an independent risk factor for failure to clear high-risk HPV [32,42,45].	Proposed based on external literature.
*Fusobacterium* spp. (e.g., *F. nucleatum*)	Not reported.	Promotes IL-4 and TGF-β1 mRNA expression, activating Treg and dendritic cells to induce an immunosuppressive phenotype [45].	Creates an immunosuppressive microenvironment that allows the virus to evade host immune clearance, facilitating persistent infection and progression to cervical cancer [32,45]	Proposed based on external literature.
*Fannyhessea vaginae* (formerly *Atopobium vaginae*)	Contributes to nucleotide biosynthesis via the inosine-5′-phosphate and 5-aminoimidazole ribonucleotide pathways [32].	Induces immune dysregulation alongside other anaerobes [32,43].	Disrupts mucosal barriers through biofilm protection, directly associated with persistent HPV infection and neoplastic progression to CIN2/3 [32].	Proposed based on external literature.
*Porphyromonas* spp. (*P. asaccharolytica*, *P. uenonis*)	Displays dose-dependent secreted proteolytic activity [37].	Degrades host coagulation factors and extracellular matrix proteins [37].	Proteolytic degradation of the host extracellular matrix serves as a primary virulence mechanism, breaking down structural barriers to facilitate persistent HPV infection [37]	Proposed based on external literature.
*Peptostreptococcus anaerobius*	Not reported.	Not reported.	Works synergistically with other dysbiotic bacteria to disrupt mucosal homeostasis, creating an environment favourable for CIN progression [32,36]	Proposed based on external literature.
*Staphylococcus* spp. (*S. aureus*, *S. haemolyticus*)	Secretes distinct toxins into the vaginal microenvironment [34,37].	Secretes immune-modulating factors [34,37].	Alters local immune surveillance and host mucosal barrier integrity, impairing viral trapping and enabling HPV infection persistence (specifically associated with alpha-9 HPV types like HPV 16) [34,37].	Proposed based on external literature.
*Corynebacterium* spp.	Binds to intercellular matrix proteins [24].	Stimulates local cytokine production [24].	Adheres to epithelial cells and competes for adhesion sites, promoting dysbiosis and facilitating CIN progression [24].	Proposed based on external literature.
*Burkholderia stagnalis*	Not reported.	Subverts natural immune functions and elicits targeted inflammation [24].	Highly prevalent across cohorts; its immune subversion and intrinsic antimicrobial resistance may facilitate the occurrence of SIL [24].	Proposed based on external literature.
Community-level/polymicrobial profile				
Anaerobic Consortia (General CST IV ^2^ Profile)	Increases L-lactic acid to D-lactic acid ratio, elevating EMMPRIN and MMP-8 [29]. Upregulates mucin-degrading enzymes while downregulating protective H_2_O_2_ [35]. Ectopic nitrogen/lipid metabolism generates carcinogenic nitrosamines [15,36]	Triggers chronic local inflammation and mucosal barrier degradation [41,43]. Skews macrophage polarisation [36].	Elevated MMP-8, EMMPRIN, and mucin-degrading enzymes compromise cervical epithelial integrity, physically facilitating HPV anchoring, replication, and oncogenic progression [29,35].	In vivo metabolic profiling [29] and metagenomic functional pathway mapping [32].

Note: The enzymatic, immune, and mechanistic actions described in this table reflect the biological rationales and hypotheses provided by the authors of the included studies (often synthesised in their respective discussion sections using external literature). These mechanisms were not necessarily validated in vivo by the primary included cohorts themselves. ^1^ Not reported: no data identified for this domain at the genus/species level among included studies. ^2^ CST IV: community state type IV, a polymicrobial (no single taxon) profile. ^3^ LPS, lipopolysaccharide. ^4^ IL, interleukin; IgA, immunoglobulin A; MMP-8, matrix metalloproteinase-8. ^5^ CIN, cervical intraepithelial neoplasia; HPV, human papillomavirus; EMMPRIN, extracellular matrix metalloproteinase inducer; TGF-β1, transforming growth factor beta; SIL, squamous intraepithelial lesion.

## Data Availability

All data generated or analysed during this study are included in this published article and its Supplementary Information Files.

## Supplementary Materials

The following supporting information can be downloaded at https://www.mdpi.com/article/10.3390/ijms27198625/s1.

## Abbreviations

The following abbreviations are used in this manuscript:

HPV	Human papillomavirus
hrHPV	High-risk human papillomavirus
CIN	Cervical intraepithelial neoplasia
CST/CSTs	Community state type/Community state types
PRISMA	Preferred Reporting Items for Systematic Reviews and Meta-Analyses
MeSH	Medical Subject Headings
JBI	Joanna Briggs Institute
LSIL	Low-grade squamous intraepithelial lesion
HSIL	High-grade squamous intraepithelial lesion
ICC	Invasive cervical cancer
NILM	Negative for intraepithelial lesion or malignancy
NSIL	No squamous intraepithelial lesion
HRCD	High-risk cervical disease
BV	Bacterial vaginosis
PCR	Polymerase Chain Reaction
rRNA/dRNA	Ribosomal ribonucleic acid/Ribosomal deoxyribonucleic acid
LC-MS	Liquid chromatography–mass spectrometry
qPCR	Quantitative polymerase chain reaction
RDB	Reverse dot blot
HC2	Hybrid Capture 2 assay
IQR	Interquartile range
ITS1	Internal transcribed spacer 1
IL	Interleukin
IgA	Immunoglobulin A
LPS	Lipopolysaccharide
TGF-β1	Transforming growth factor beta 1
EMMPRIN	Extracellular matrix metalloproteinase inducer
MMP-8	Matrix metalloproteinase-8
NK	Natural killer (cells)
TIGIT	T cell immunoglobulin and ITIM domain
ROS	Reactive oxygen species
aOR	Adjusted odds ratio
OR	Odds ratio
AUC	Area under the curve
PPV	Positive predictive value
LDA	Linear discriminant analysis
GLM	Generalised linear model
OTU	Operational taxonomic unit
LEfSe	Linear discriminant analysis effect size
PTS	Phosphotransferase system
SCFA/SCFAs	Short-chain fatty acid/Short-chain fatty acids
PE	Phosphatidylethanolamine
PI	Phosphatidylinositol
