# Supplementary material for: Vaginal Microbial Risk Factors Associated with Human Papillomavirus-Induced Cervical Intraepithelial Neoplasia: A Systematic Review
Source: Int J Mol Sci. 2026 Sep 26;27(19):8625. doi: 10.3390/ijms27198625 (PMC13665079; doi:10.3390/ijms27198625)
Supplement: Supplementary file 1 [file ijms-27-08625-s001.zip › Figure S1 Reported P-Values for Alpha and Beta Diversity by Study.pdf]

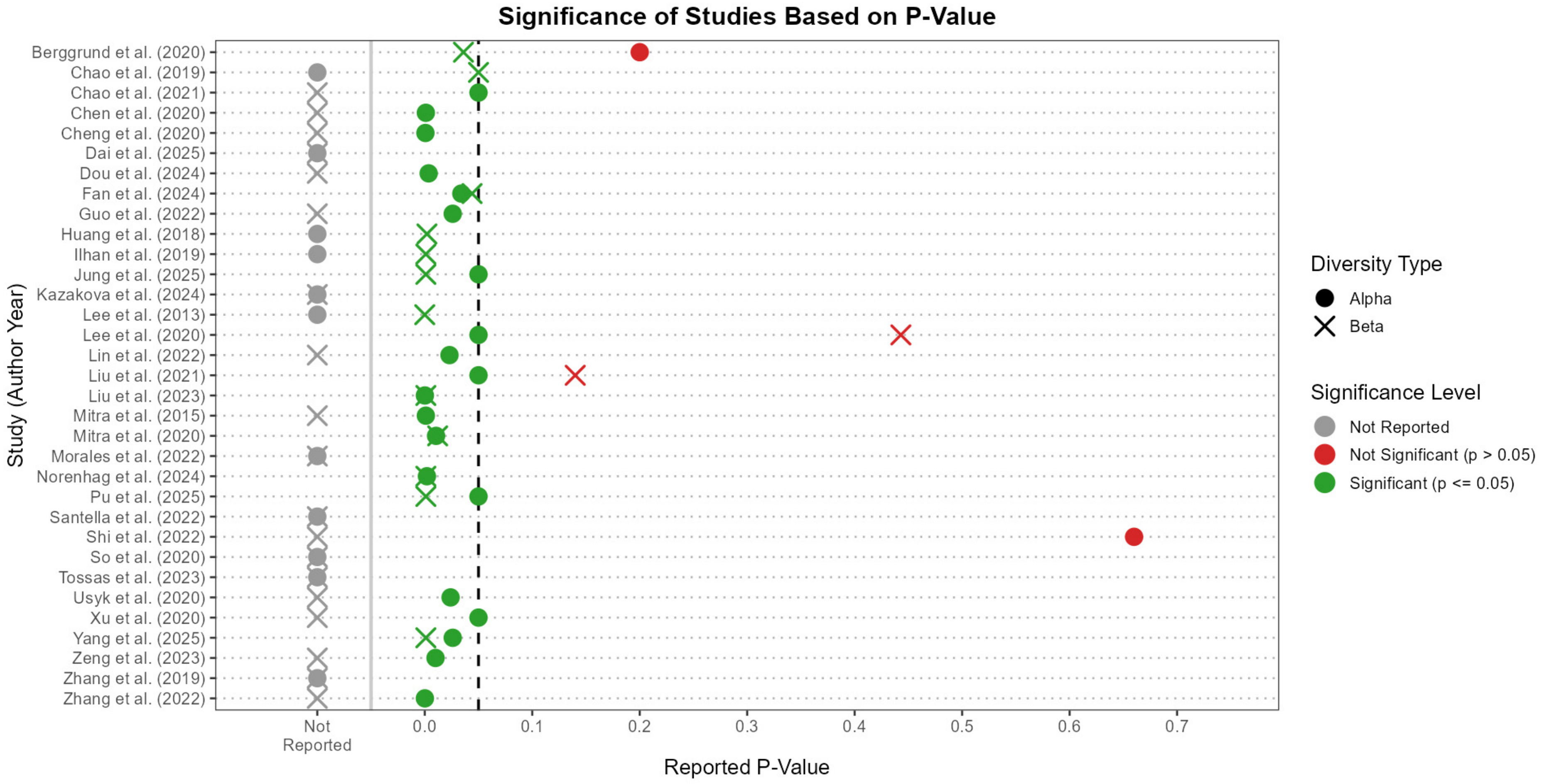

Figure S1: Reported P-Values for Alpha and Beta Diversity by Study

Dot plot illustrating the specific numeric p-values reported by each study for alpha diversity (circles) and beta diversity (crosses) metrics. Green markers represent statistically significant findings ($p \leq 0.05$), while red markers represent non-significant findings

($p > 0.05$). The vertical dashed black line denotes the standard significance threshold at $p = 0.05$. Grey markers located to the left of the solid grey dividing line indicate studies where specific numeric p-values were not explicitly reported.
