# Supplementary material for: Vaginal Microbial Risk Factors Associated with Human Papillomavirus-Induced Cervical Intraepithelial Neoplasia: A Systematic Review
Source: Int J Mol Sci. 2026 Sep 26;27(19):8625. doi: 10.3390/ijms27198625 (PMC13665079; doi:10.3390/ijms27198625)
Supplement: Supplementary file 1 [file ijms-27-08625-s001.zip › Figure S2 Alpha Diversity Directions Across Included Studies.pdf]

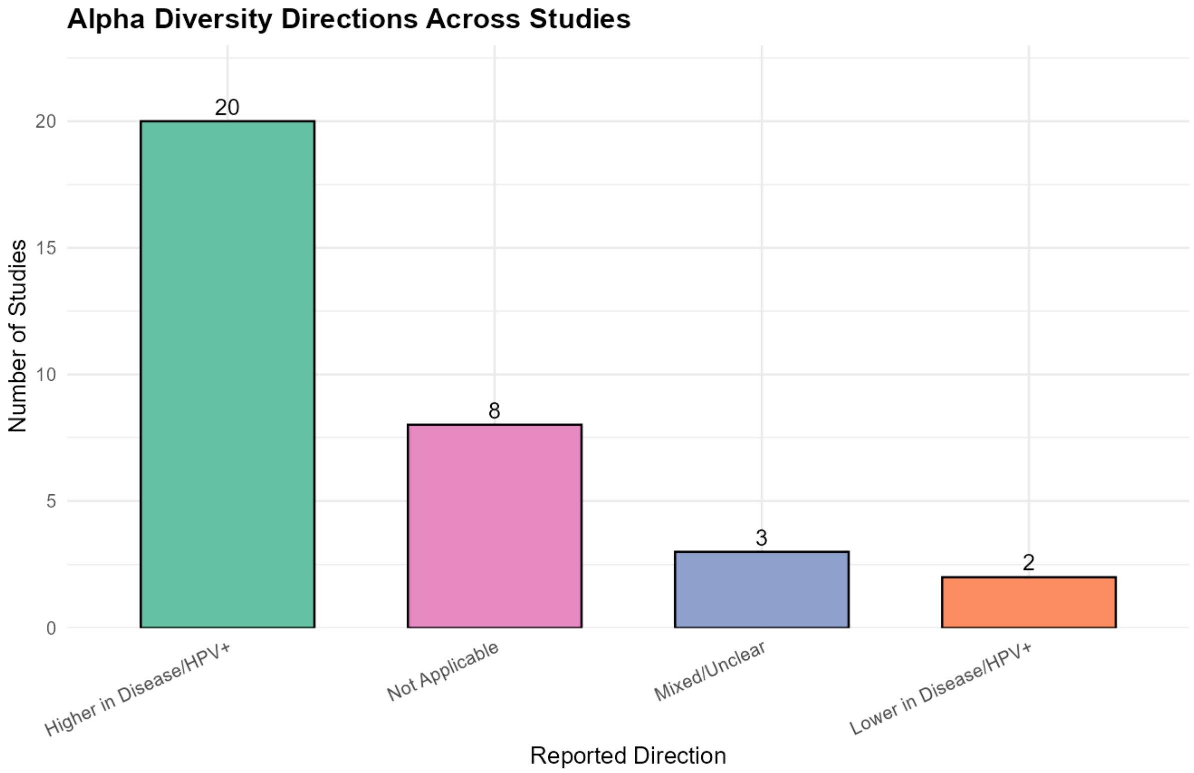

Figure S2: Alpha Diversity Directions Across Included Studies

Bar chart summarizing the reported direction of alpha diversity shifts in disease or HPV-positive groups compared to controls across the reviewed literature. Bars indicate the total number of studies for each reported outcome, with the most frequent observation being higher alpha diversity in disease/HPV-positive states.
