# Supplementary material for: Vaginal Microbial Risk Factors Associated with Human Papillomavirus-Induced Cervical Intraepithelial Neoplasia: A Systematic Review
Source: Int J Mol Sci. 2026 Sep 26;27(19):8625. doi: 10.3390/ijms27198625 (PMC13665079; doi:10.3390/ijms27198625)
Supplement: Supplementary file 1 [file ijms-27-08625-s001.zip › Figure S3 Beta Diversity Significance Outcomes.pdf]

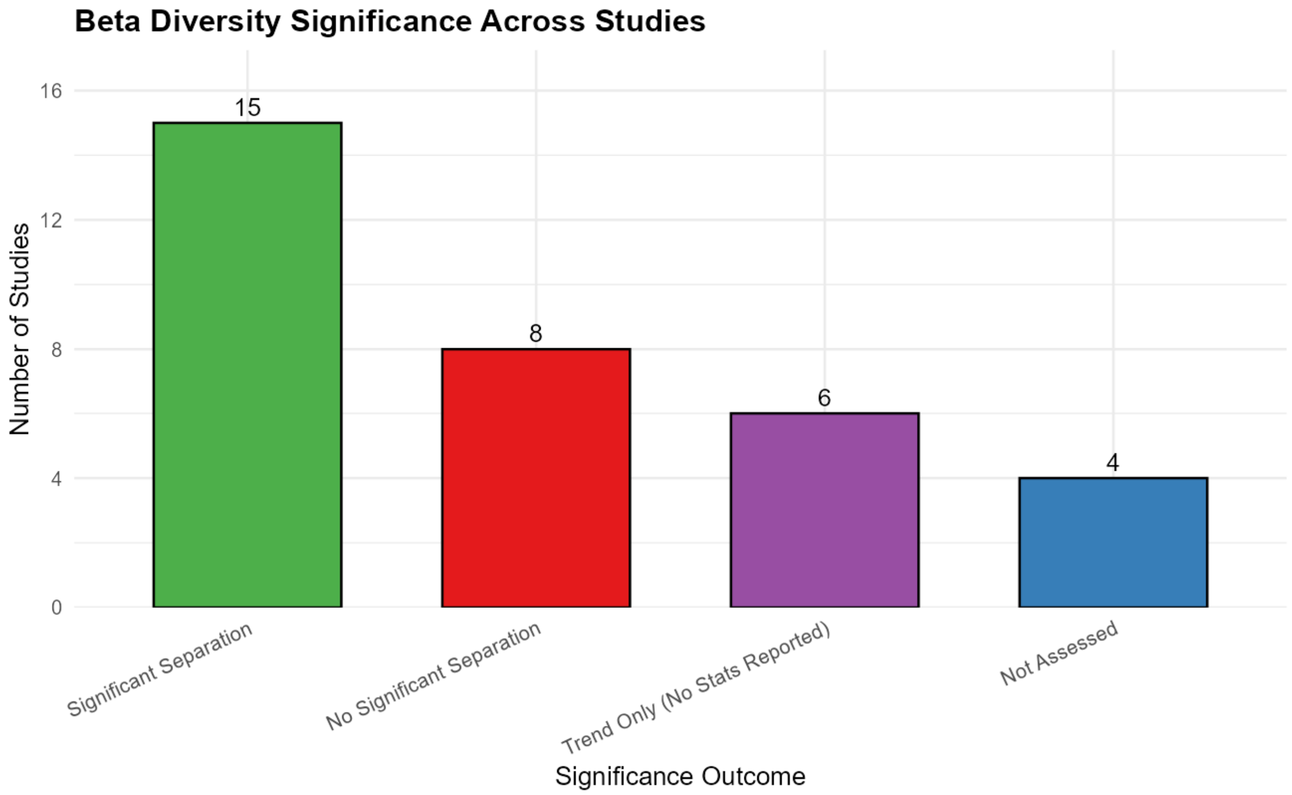

Figure S3: Beta Diversity Significance Outcomes

Bar chart summarizing the qualitative statistical outcomes for beta diversity across the reviewed studies. Categories indicate whether studies found significant community separation, no significant separation, a visible trend without reported statistics, or did not assess beta diversity.
