# Supplementary material for: Vaginal Microbial Risk Factors Associated with Human Papillomavirus-Induced Cervical Intraepithelial Neoplasia: A Systematic Review
Source: Int J Mol Sci. 2026 Sep 26;27(19):8625. doi: 10.3390/ijms27198625 (PMC13665079; doi:10.3390/ijms27198625)
Supplement: Supplementary file 1 [file ijms-27-08625-s001.zip › Table S2. Full Search Strategies by Database.pdf]

**Table S2.** Full Search Strategies by Database

Search strategies, MeSH terms, text words, and concepts of records retrieved from PubMed, Web of Science, ProQuest, Scopus, and CINAHL.

| | Concept 1 | Concept 2 | Concept 3 |
|---|---|---|---|
| MeSH Terms | Microbiota OR “Vaginosis, Bacterial” OR “Bacteria, Anaerobic” | Papillomaviridae OR “Papillomavirus Infections” | “Uterine Cervical Dysplasia” OR “Uterine Cervical Neoplasms” |
| Text word | Microbiota OR “Vaginosis, Bacterial” OR “Bacteria, Anaerobic” OR microbiome OR “microbial community” OR “vaginal microbiome” OR vaginome OR “vaginal microbiota” OR “vaginal biota” OR “vaginal flora” OR “vaginal microflora” OR “vaginal bacteria” OR “vaginal microbial community” OR "vaginal microbial ecosystem” OR “vaginal microbial profile” OR “microbial risk factors” OR “community state types” OR CST OR “abnormal vaginal flora” | Papillomaviridae OR “Papillomavirus Infections” OR<br>HPV OR “human papillomavirus” OR “human papilloma virus” | “Uterine Cervical Dysplasia” OR “Uterine Cervical Neoplasms” OR<br>“cervical intraepithelial neoplasia” OR CIN OR “cervical dysplasia” OR “cervix dysplasia” OR “cervical precancer” OR “cervix uteri dysplasia” OR “cervical lesions” OR “precancerous cervical lesions” OR “cervical neoplasia” |

Note: This table details the specific search strings, Medical Subject Headings (MeSH), and keywords applied to each electronic database. Searches were conducted for literature published up to October 2025 and were restricted to peer-reviewed, full-text articles published in English.
