# Supplementary material for: Vaginal Microbial Risk Factors Associated with Human Papillomavirus-Induced Cervical Intraepithelial Neoplasia: A Systematic Review
Source: Int J Mol Sci. 2026 Sep 26;27(19):8625. doi: 10.3390/ijms27198625 (PMC13665079; doi:10.3390/ijms27198625)
Supplement: Supplementary file 1 [file ijms-27-08625-s001.zip › Table S3. Risk of Bias Assessment for Included Studies.pdf]

Table S3. Risk of Bias Assessment for Included Studies

a) Summary of quality assessments (cohort studies)

**JBI Checklist / References**	**Cohort Studies**											**Quality Score**	**Risk of Bias**
	Q1. Were the two groups similar and recruited from the same population?	Q2. Were the exposures measured similarly to assign people to both exposed and unexposed groups?	Q3. Was the exposure measured in a valid and reliable way?	Q4. Were confounding factors identified?	Q5. Were strategies to deal with confounding factors stated?	Q6. Were the groups/participants free of the outcome at the start of the study (or at the moment of exposure)?	Q7. Were the outcomes measured in a valid and reliable way?	Q8. Was the follow up time reported and sufficient to be long enough for outcomes to occur?	Q9. Was the follow up complete, and if not, were the reasons to loss to follow up described and explored?	Q10. Were strategies to address incomplete follow up utilized?	Q11. Were appropriate statistical analysis used?		
Berggrund et al. (2020) [39]	Y	Y	Y	N	N	Y	Y	Y	Y	N	Y	8/11 (72.7)	Low
Mitra et al. (2020) [40]	Y	Y	Y	Y	Y	Y	Y	Y	Y	Y	Y	11/11 (100.0)	Low
Shi (2022) [41]	Y	Y	Y	Y	Y	Y	Y	Y	Y	N	Y	10/11 (90.9)	Low
Tossas et al. (2023) [42]	Y	Y	Y	Y	Y	Y	Y	Y	U	N	Y	9/11 (81.8)	Low
Usyk et al. (2020) [43]	Y	Y	Y	Y	Y	Y	Y	Y	U	N	Y	9/11 (81.8)	Low
Zeng et al. (2023) [44]	Y	Y	Y	U	U	Y	Y	Y	U	U	Y	7/11 (63.6)	Moderate

b) Summary of quality assessments (cross-sectional studies)

JBI Checklist / References	Cross-sectional Studies								Quality Score	Risk of Bias
	Q1. Were the criteria for inclusion in the sample clearly defined?	Q2. Were the study subjects and the setting described in detail?	Q3. Was the exposure measured in a valid and reliable way?	Q4. Were objective, standard criteria used for measurement of the condition?	Q5. Were confounding factors identified?	Q6. Were strategies to deal with confounding factors stated?	Q7. Were the outcomes measured in a valid and reliable way?	Q8. Was appropriate statistical analysis used?		
Chao et al. (2020) [13]	Y	Y	Y	Y	U	N	Y	Y	6/8 (75.0)	Low
Chao et al. (2021) [14]	Y	Y	Y	Y	U	N	Y	Y	6/8 (75.0)	Low
Chen et al. (2020) [15]	Y	Y	Y	Y	U	N	Y	Y	6/8 (75.0)	Low
Cheng et al. (2020) [16]	Y	Y	Y	Y	Y	Y	Y	Y	8/8 (100.0)	Low
Dai et al. (2025) [17]	Y	Y	Y	Y	Y	Y	Y	Y	8/8 (100.0)	Low
Dou et al. (2024) [18]	Y	Y	Y	Y	U	N	Y	Y	6/8 (75.0)	Low
Fan et al. (2024) [19]	Y	Y	U	Y	U	N	Y	Y	6/8 (75.0)	Low
Guo et al. (2022) [20]	Y	Y	Y	Y	Y	Y	Y	Y	8/8 (100.0)	Low
Huang et al. (2018) [21]	Y	Y	U	Y	N	N	Y	Y	5/8 (62.5)	Moderate
Ilhan et al. (2019) [22]	Y	Y	Y	Y	Y	Y	Y	Y	8/8 (100.0)	Low
Jung et al. (2025) [23]	Y	Y	Y	Y	U	N	Y	Y	6/8 (75.0)	Low
Kazakova et al. (2024) [24]	Y	Y	Y	Y	Y	Y	Y	Y	8/8 (100.0)	Low
Lee et al. (2013) [25]	Y	Y	Y	Y	U	U	Y	Y	6/8 (75.0)	Low
Lee et al. (2020) [26]	Y	Y	Y	Y	N	N	Y	Y	6/8 (75.0)	Low
Lin et al. (2022) [27]	Y	Y	U	Y	U	N	Y	Y	5/8 (62.5)	Moderate
Liu, Li, et al. (2023) [28]	Y	Y	Y	Y	Y	Y	Y	Y	8/8 (100.0)	Low
Liu, Wang, et al. (2023) [29]	Y	Y	Y	Y	N	N	Y	Y	6/8 (75.0)	Low
Mitra et al. (2015) [30]	Y	Y	Y	U	U	N	Y	Y	5/8 (62.5)	Moderate
Morales et al. (2022) [31]	Y	Y	Y	Y	Y	Y	Y	Y	8/8 (100.0)	Low
Norenhag, J. (2024) [32]	Y	Y	Y	Y	Y	Y	Y	Y	8/8 (100.0)	Low
Pu et al. (2025) [33]	Y	Y	Y	Y	U	N	Y	Y	6/8 (75.0)	Low
Santella et al. (2022) [34]	Y	Y	Y	Y	N	N	Y	Y	6/8 (75.0)	Low
So et al. (2020) [35]	Y	Y	Y	Y	N	N	Y	Y	6/8 (75.0)	Low
Xu et al. (2023) [36]	Y	Y	Y	Y	U	N	Y	Y	6/8 (75.0)	Low
Yang et al. (2025) [37]	Y	Y	Y	Y	U	N	Y	Y	6/8 (75.0)	Low
Zhang et al. (2018) [38]	Y	Y	Y	Y	N	N	Y	Y	6/8 (75.0)	Low
Zhang et al. (2022) [39]	Y	Y	Y	Y	Y	Y	Y	Y	8/8 (100.0)	Low

Note: Methodological quality was evaluated using the Joanna Briggs Institute (JBI) critical appraisal checklists for cohort studies (11 criteria) and analytical cross-sectional studies (8 criteria). Ratings for each checklist item are indicated as Y (Yes), N (No), or U (Unclear). Quality scores reflect the proportion of criteria met, with studies classified as having a Low or Moderate risk of bias based on their final percentages.
