# Supplementary material for: Vaginal Microbial Risk Factors Associated with Human Papillomavirus-Induced Cervical Intraepithelial Neoplasia: A Systematic Review
Source: Int J Mol Sci. 2026 Sep 26;27(19):8625. doi: 10.3390/ijms27198625 (PMC13665079; doi:10.3390/ijms27198625)
Supplement: Supplementary file 1 [file ijms-27-08625-s001.zip › Table S4. Microbial taxa, diversity and CST findings associated with HPV and CIN.pdf]

Table S4. Microbial taxa, diversity and CST findings associated with HPV and CIN

**References**	**Increased taxa**	**Decreased taxa**	**Alpha Diversity** [2,3]	**Beta Diversity** [12,3]	**CST/dominance pattern** [1,2]	**Persistence / progression link** [1,2,3]	**Diagnostic accuracy** [2,3]
Chao et al. (2020) [13]	*Actinobacteria, Proteobacteria, Bacteroidetes*	*Firmicutes, Lactobacillus*	NS (Shannon; Persistent hrHPV vs Incident hrHPV p=0.62, Persistent hrHPV vs HPV- p=0.41); richness trend decrease in persistent	Significant: Persistent hrHPV vs Incident hrHPV (ANOSIM p=0.016), Persistent hrHPV vs HPV- (p=0.04); not Incident hrHPV vs HPV-	Not formally CST-classified	*P. bivia* + *P. disiens* combo tracked with persistence gradient	*P. bivia*>0.055%+P. disiens<0.022% → 100% sens/66.7% spec for CIN2+
Chao et al. (2021) [14]	*Bacteroidetes (*LEfSe*)*	*Firmicutes*	Increased in HSIL (Shannon; HSIL vs HPV+ p=0.0065, HSIL vs HPV- p=0.025)	Significant all pairwise (weighted UniFrac, p<0.05)	Not CST-classified; NLD pattern in HSIL	HSIL shows greatest dysbiosis vs HPV+ w/o disease	*Stenotrophomonas/ Faecalibacterium/ Bifidobacterium* → PPV 76–83%
Chen et al. (2020) [15]	*Gardnerella, Atopobium, Prevotella, Sneathia*	*Lactobacillus, Firmicutes*	Increase progressively Normal→Cancer (OTU, Shannon)	Significant Normal vs HPV/LSIL/Cancer; NOT sig. LSIL vs HSIL or HSIL vs Cancer	CST IV: 41%(HPV)→35%(LSIL)→57%(HSIL)→89%(Cancer)	Major shift at Normal→HPV and HPV→HSIL; LSIL→HSIL→Cancer NS	Not formal
Cheng et al. (2020) [16]	BVAB1<br>BVAB2<br>Sneathia<br>Prevotella<br>Megasphaera	*Lactobacillus crispatus*	Significant ($p < 0.001$ for Shannon, Chao1, and Faith's PD). Significantly higher in HPV+, especially in multi-type infections ($p < 0.001$) and oncogenic HPV infections ($p < 0.05$).	Not significant.	Non-*Lactobacillus* dominant profile was 2× more common in HPV+ (Fisher's exact $p = 0.011$).<br><br>High risk with non-*Lactobacillus* dominance (Adjusted OR = 2.0 for oncogenic HPV; OR = 2.4–2.5 for multiple HPV types).	Anaerobic dysbiosis (*BVABs, Prevotella, Sneathia*) strongly associated with oncogenic and persistent HPV subtypes.	Specific bacterial biomarkers (*notably BVAB1, Sneathia, Prevotella*) proposed as risk stratification biomarkers, but formal sensitivity/specificity (AUC/ROC) curves were not generated

Table S4. *Cont.*

References	Increased taxa	Decreased taxa	Alpha Diversity [2,3]	Beta Diversity [12,3]	CST/dominance pattern [1,2]	Persistence / progression link [1,2,3]	Diagnostic accuracy [2,3]
Dai et al. (2025) [17]	*Fannyhessea, Gardnerella (*non-*Lactobacillus*-dominated microbiota*)*	*Lactobacillus (*non-*Lactobacillus*-dominated microbiota)	Increase in HPV+ (Shannon p=0.0036, Simpson p=0.02); Chao1/ACE NS ; NS between CIN1/CIN2+	Slight separation (weighted UniFrac PCoA); ANOSIM p not stated	LD 82.6%(CIN1) vs 70%(CIN2+), NS	No sig. CST/diversity shift CIN1→CIN2+; shift is metabolomic/transcriptomic	None (exploratory mediation only)
Dou et al. (2024) [18]	*Gardnerella vaginalis, Aerococcus, Actinobacteria*	*Lactobacillus, Firmicutes*	Increased Shannon/Simpson (p=0.0036/0.02)	Slight separation (weighted UniFrac PCoA); ANOSIM p not stated	CST IV-like features increased in HPV+ (not formally classified)	N/A (cross-sectional only)	G. vaginalis LDA>3.5; no formal AUC
Fan et al. (2024) [19]	*Dialister, Peptoniphilus, Proteobacteria*	*Lactobacillus, Cyanobacteria*	Increased in CC only vs hrHPV+/CIN (p=0.034, p=0.019); Chao1 NS	Significant but modest (ANOSIM R=0.046, p=0.044)	Not CST-classified	Major dysbiosis appears only at CC stage, not CIN	RF AUC: Normal 0.75, CIN 0.71, CC 0.68, hrHPV 0.51
Guo et al. (2022) [20]	*Megasphaera, Gardnerella, Fannyhessea, Prevotella*	*Enterococcus*	Increased HSIL vs LSIL (Shannon p=0.026); NS earlier stages	SIL severity > HPV status in driving separation (PERMANOVA)	LD vs NLD; NLD 17.9%(HPV+HSIL) vs 10%(HPV−)	SIL severity (not HPV status) drives CVM shift; biggest jump at LSIL→HSIL	RF top OTUs; no formal AUC
Huang et al. (2018) [21]	Type-specific rare taxa (*Oribacterium*-HPV16; *Motilibacter*-HPV52)	*L. iners* (HPV58), *L. crispatus*	Increased in hrHPV+ vs Nor (Chao1/ACE/Shannon/InvSimpson, all p<0.05); richness↑ but species count dissociation (HSIL genus 268 vs LSIL 264, but species 977 vs 1,057)	Nor separates from LSIL/HSIL (NMDS); ANOSIM sig within-type: 16SIL p=0.001, 52SIL p=0.001, 58SIL p=0.002	4 clusters (Cluster I=crispatus, III=iners, II/IV=BV-like)	Rare/type-specific taxa, not diversity, drive type acquisition	PLS-DA VIP; no clinical AUC

Table S4. *Cont.*

References	Increased taxa	Decreased taxa	Alpha Diversity [2,3]	Beta Diversity [12,3]	CST/dominance pattern [1,2]	Persistence / progression link [1,2,3]	Diagnostic accuracy [2,3]
Ilhan et al. (2019) [22]	*Gardnerella, Prevotella, Streptococcus, Atopobium, Sneathia*	*Lactobacillus*	Metabolite diversity U-shaped (not microbial): non-linear trend (p=0.0004); ICC highest (decreased then increased in ICC)	PCA: ICC distinct from all groups (p=0.006); HSIL distinct from Ctrl HPV− on PC1 only (p=0.046)	LD vs NLD (binary); NLD in dysplasia/ICC	Metabolome driven more by cancer / inflammation than microbiome per se	Metabolite AUC up to 0.9+ (not taxa-based)
Jung et al. (2025) [23]	*Fannyhessea vaginae, Anaerococcus, E. coli*	*Lactobacillus* (esp. *crispatus*)	NS by HPV type; increased with lesion severity/age	NMDS separates by CST not HPV type; PERMANOVA NS by HPV type overall (p=0.308), sig by lesion (p=0.001)	CST I/III/IV-B/IV-C; CST IV increased with severity; HPV16/18 lacked CST I at high load	Progressive *Lactobacillus* loss with severity in HPV16/18	None formal
Kazakova et al. (2024) [24]	*Streptococcus* (3.7×), *Corynebacterium* (2.8×)	not quantified	Not applicable (culture-based, no sequencing)	Not applicable	Not assessed	HSIL association only, single time point	Prevalence ratios only, no AUC
Lee et al. (2013) [25]	*Fusobacteria, Sneathia* (>3× in HR-HPV)	*Lactobacillus* (47% vs 77%)	Increased in diversity; HPV+ premenopausal vs HPV−; Inc further in HPV+ discordant twins ($P<10^{-8}$)	MZ twins more similar to each other (P<0.05); HPV+ vs HPV− discordant twins $P<10^{-8}$	Cluster III (iners) vs I (crispatus), NS by HPV (p=0.17)	Discordant MZ twin design controls genetics. *L. iners* decreased significant (p=0.03)	None formal
Lee et al. (2020) [26]	*Streptococcus spp.* (5 species), *Gardnerella*	*L. johnsonii*	NS overall CIN1− vs CIN2+; CST 4-A highest, CST 4-B lowest (ANOVA p<0.001 across CST) (PCA p=0.443)	NS CIN1− vs CIN2+ (PCA p=0.443); significant CST clustering (Adonis p<0.001)	CST3/4-A/4-B/CST* all NS by CIN grade	*L. iners* top RF predictor of CIN2+	33-species RF: AUC 0.952, sens 100%, spec 85.7%

Table S4. *Cont.*

References	Increased taxa	Decreased taxa	Alpha Diversity [2,3]	Beta Diversity [12,3]	CST/dominance pattern [1,2]	Persistence / progression link [1,2,3]	Diagnostic accuracy [2,3]
Lin et al. (2022) [27]	*Gardnerella, Prevotella (Actinobacteria* increased)	*Lactobacillus* (95.7%→62.9%)	Increased in HPV+ (Shannon F=6.14 p=0.023; Simpson F=9.49 p=0.006); Chao/ACE NS	Not formally reported with PCoA/NMDS stats	GV-dominant vs Lactobacillus-dominant; GV assoc. HR-HPV 72.7%	N/A	SNA enzyme assay: p=0.004 (HPV), p=0.041 (CIN); no AUC
Liu, Li, et al. (2023) [28]	*Gardnerella, Prevotella, Sneathia, Dialister* (12 genera)	*Lactobacillus*	Increased progressively stepwise across non-BV&HPV− < non-BV&HPV+ < BV&HPV− < BV&HPV+ (sig, all indices except Pielou's)	Significant separation all 4 groups (Adonis p<0.001); NS between noncases/CIN1/CIN2+	Not CST-labeled; BV = largest driver (Adonis)	BV/HPV-enriched taxa peak at CIN1 then decline at CIN2+ (HPV becomes dominant driver)	None formal
Liu, Wang, et al. (2023) [29]	*Gardnerella, Dialister, Prevotella, Ureaplasma*	*Lactobacillus, Pseudomonas*	Increased progressively NSIL→CIN3 (Shannon p<0.001)	Not significant (PCoA p=0.14) diversity ↑ without compositional shift	Not formally CST-classified; CST IV concept discussed	Clear step-wise gradient NSIL→CIN3	None formal (LEfSe only)
Mitra et al. (2015) [30]	*Sneathia sanguinegens, Peptostreptococcus*	*L. jensenii, L. coleohominis*	Increased with severity, not stat. sig across disease groups; sig higher CST IV vs CST I (p<0.001) and CST III (p<0.01) (highest ICC)	PCA species-level 3 clusters (PC1=56.2%); no formal PERMANOVA p reported	CST IV: 10%→21%→27%→40% (stepwise, NS)	Consistent stepwise trend, underpowered	None formal
Morales et al. (2022) [31]	*Roseburia, Prevotella, Dialister, Gardnerella*	*Lactobacillus crispatus*	Not formally reported	Not formally reported (Jensen-Shannon used for CST only)	CST IV OR 1.96 (any HPV); NS for hrHPV specifically	N/A	MB score (30 taxa) AUC 0.80

Table S4. *Cont.*

References	Increased taxa	Decreased taxa	Alpha Diversity [2,3]	Beta Diversity [12,3]	CST/dominance pattern [1,2]	Persistence / progression link [1,2,3]	Diagnostic accuracy [2,3]
Norenhag, J. (2024) [32]	*Gardnerella vaginalis, Fannyhessea vaginae, Aerococcus christensenii, Peptoniphilus lacrimalis*	*Lactobacillus crispatus*	Significant (Shannon $p < 0.002$, inverse Simpson $p < 0.01$, species richness $p < 0.001$). Significantly higher in dysplasia vs controls, but Not Significant between LSIL and HSIL/cancer ($p > 0.05$) or between CIN2 and CIN3 ($p > 0.05$)	Significant (PERMANOVA $p < 0.001, R^2 = 0.022$) between healthy controls and dysplasia. However, Not Significant between LSIL and HSIL/cancer ($p > 0.05$) or between CIN2 and CIN3 ($p > 0.05$)	Controls dominated by CST-I (42.4%), followed by CST-IV (31.0%) and CST-III (24.3%)<br><br>Dysplasia dominated by CST-IV (44.6%: IV-B 39.5%), followed by CST-III (26.0%) and CST-I (24.3%) ($p = 0.0047$)	Depletion of *L. crispatus* and expansion of peptidoglycan/nucleotide-synthesizing anaerobes (*G. vaginalis, F. vaginae, P. lacrimalis*) create a pro-oncogenic niche promoting dysplasia	Dysbiotic taxa (*P. lacrimalis, A. christensenii, G. vaginalis, F. vaginae*) distinguished dysplasia from controls; self-reported abnormal discharge correlated significantly with CST-IV dysbiosis ($p = 0.047$), but formal ROC diagnostic performance was not evaluated.
Pu et al. (2025) [33]	*L. iners (paradoxical), P. bivia, G. vaginalis*	*L. crispatus*	Not formally calculated (qualitative only)	Not formally calculated (k-means used instead)	Cluster II anaerobes: tissue 0%→100%(NH)→88%(CIN)	*L. iners* rises then falls (HPV→CIN transition)	Combined micro+metabolite AUC up to 1.0
Santella et al. (2022) [34]	*Enterococcus, Staphylococcus, Gardnerella, Morganella, Proteobacteria*	*Lactobacillus* (99.2%→84.5%)	NS (Shannon p=0.664, Chao1 p=0.074, Simpson p=0.987)	NS (PERMANOVA R^2=0.0153, p=0.34)	Not CST-classified	N/A	Non-formal
So et al. (2020) [35]	*G. vaginalis, Prevotella spp., Dialister, Atopobium*	*L. crispatus*	Increased HPV+ vs HPV− (p=0.002)<br><br>NS overall by CIN3; race × CIN3 interaction significant	Significant by race (p≤0.001) and CIN3 (p=0.003)	Not CST-classified	Species-level ORs for CIN2/3+cancer	6-taxa ORs (e.g., P. buccalis OR=11.0); no combined AUC

Table S4. *Cont.*

References	Increased taxa	Decreased taxa	Alpha Diversity [2,3]	Beta Diversity [12,3]	CST/dominance pattern [1,2]	Persistence / progression link [1,2,3]	Diagnostic accuracy [2,3]
Xu et al. (2023) [36]	*Halomonas, Fusobacterium, Enterococcus*	*Lactobacillus*	Significant across 4 groups (Chao1 p=0.048); CIN2+ vs HPV− p=0.026; HPV+noCIN vs HPV− NS (p=0.103)	Not explicitly reported sig/NS; hierarchical clustering/MDS used	Not CST-classified	N/A	10-OTU RF model AUC 89.7% (discovery), 81.9% (validation)
Yang et al. (2025) [37]	*Fannyhessea vaginae, Sneathia vaginalis, Aerococcus*	*Lactobacillus* (varies by CST)	Increase in Hr-HPV16/18 (Chao1/ACE p<0.05); Shannon/Simpson NS; clearance vs persistence Chao1/ACE sig (p=0.046/0.04)	NS (PCoA unweighted UniFrac, no clear separation)	VALENCIA: CST IV subtypes ~69.6% of cohort; CST IV-B linked high-grade CIN	Stage-dependent taxa (CIN1 vs CIN2+ markers differ)	*F. vaginae+P. vulgatus* combo PPV 90%
Zhang et al. (2018) [38]	*L. crispatus* (paradoxically increased CIN2+), *S. agalactiae, B. fragilis*	*L. delbrueckii*	Significant increase in HC→HPV→CIN (Shannon/Chao1/Faith's PD, all P<0.0001)	Separates into Lactobacillus-dominated vs non-Lactobacillus clusters; no clear HPV/CIN subgroup separation	Not CST-classified	Mediation analysis: taxa have opposing direct/indirect (via HPV) effects on CIN	None (mediation ORs only)
Zhang et al. (2022) [39]	*Streptococcus, Prevotella, Chlamydia*	*Bifidobacterium, Ralstonia, L. crispatus*	Increase progressively (p<0.0001)	Significantly higher in HPV-positive and CIN groups than healthy controls (Shannon, Chao1, Faith's PD; all *P* < 0.05).	No distinct clustering between HPV/CIN groups and healthy controls; separation mainly by Lactobacillus-dominated status (Bray–Curtis PCoA).	HPV infection, only significant risk factor for CIN (OR 4.14)	Non-formal

Table S4. *Cont.*

References	Increased taxa	Decreased taxa	Alpha Diversity [2,3]	Beta Diversity [12,3]	CST/dominance pattern [1,2]	Persistence / progression link [1,2,3]	Diagnostic accuracy [2,3]
Berggrund et al. (2020) [40]	*Gardnerella, Prevotella, Atopobium, Sneathia, Streptococcus* (dysbiotic clusters)	*Lactobacillus* (Cluster1/2)	NS between HPV-neg/transient/persistent, baseline or follow-up (all p>0.2)	Cluster-based only; baseline dysbiotic cluster freq nominal p=0.0173, lost sig after correction (q=0.0519)	4 k-means clusters; persistent higher transition rate (34% vs 19%, p=0.036)	Longitudinal: instability of microbial profile (not static dysbiosis) marks persistence	Non-formal
Mitra et al. (2020) [41]	*Prevotella timonensis, Megasphaera, Gardnerella,* BVAB1	*Lactobacillus*	Richness sig increase in non-regressors at 12mo (p=0.0105) and 24mo (p=0.0105); Inv. Simpson trend NS (p=0.0641)	Sig. composition diff regressors vs non-regressors at 12mo and 24mo (HCA, chi-sq p<0.05)	CST IV aOR 3.85 (12mo) for non-regression	True longitudinal evidence: *Lactobacillus*-depleted VMB predicts non-regression	aOR 3.56 (Lactobacillus-depleted, 12mo)
Shi (2022) [42]	*L. iners* (enriched uncleared)	*Enterococcus* ASV_62 (enriched cleared)	Increased in HPV+ (Shannon 1.06 vs 2.23, p=0.002); trend across histopath groups NS	Visual separation on PCoA (PC1=42.3%); no PERMANOVA p reported	CST NS (p=0.216)	Longitudinal: *L. iners* predicts non-clearance in LSIL subgroup (aOR 3.94)	aOR 3.34 overall for *L. iners* high abundance
Tossas et al. (2023) [43]	*Suboptimal VMB taxa (Gardnerella, Atopobium, Ca. Lachnocurva)*	*L. crispatus, L. gasseri, L. jensenii*	V1: NS (p=0.52). V2: significant rising trend clearance→persistence→progression (p=0.024)	4 CSTs by hierarchical clustering; PCoA/NMDS stats not reported in main text	Optimal 9%(CIN3) vs 19%(<CIN3); race modifies risk	Race modifies VMB–CIN3 relationship; optimal VMB not protective in non-Latina black	Suboptimal VMB OR 2.91 for CIN3
Usyk et al. (2020) [44]	*Gardnerella* (V1, LDA>4), *Prevotella, Porphyromonas*	*L. iners* (clearance marker), *Lactobacillus genus*	V2 Shannon increased trend (p=0.024)<br><br>Increase in HRCD vs HC (Shannon/Simpson p<0.05); Ace NS	NMDS/PCoA performed. Failed to distinguish groups visually	4 CSTs; *G. vaginalis*-CST 17.4%	Longitudinal + mediation: G. vaginalis → increased V2 diversity → CIN2+ (ACME p=0.04)	Lactobacillus OR=0.41; V2 Shannon OR=1.19

Table S4. *Cont.*

References	Increased taxa	Decreased taxa	Alpha Diversity [2,3]	Beta Diversity [12,3]	CST/dominance pattern [1,2]	Persistence / progression link [1,2,3]	Diagnostic accuracy [2,3]
Zeng et al. (2023) [45]	*Atopobium, Sphingomonas, S. amnii* (HPV+); *Fusobacterium/Bacteroides/Neisseria/ Helicobacter* (persistent)	*L. gasseri, A. vaginae, P. bivia*	Increased richness (Chao1/ACE) in Hr-HPV16/18, NOT Shannon (dissociation)<br><br>NS between CIN1− and CIN2+	NS (PCoA weighted UniFrac)	Not CST-classified	Partial longitudinal: persistent group had increased richness vs cleared (p=0.046)	None formal (LEfSe only)

Note: Reference numbers in this document correspond to the reference list in the main manuscript.

[1] BV, bacterial vaginosis; CST, community state type; CVM, cervicovaginal microbiome; LD, *Lactobacillus*-dominated; NLD, non-*Lactobacillus*-dominated; VMB, vaginal microbiome.

[2] CC, cervical cancer; CIN, cervical intraepithelial neoplasia; HC, healthy control; HPV, human papillomavirus; hrHPV / HR-HPV, high-risk human papillomavirus; HSIL, high-grade squamous intraepithelial lesion; ICC, invasive cervical carcinoma; LSIL, low-grade squamous intraepithelial lesion; NSIL, non-squamous intraepithelial lesion.

[3] aOR, adjusted odds ratio; AUC, area under the curve; LDA, linear discriminant analysis; LEfSe, linear discriminant analysis effect size; NMDS, non-metric multidimensional scaling; NS, not significant; OR, odds ratio; OTU, operational taxonomic unit; PCA, principal component analysis; PCoA, principal coordinate analysis; PERMANOVA, permutational multivariate analysis of variance; PPV, positive predictive value; RF, random forest.
