# Supplementary material for: Vaginal Microbial Risk Factors Associated with Human Papillomavirus-Induced Cervical Intraepithelial Neoplasia: A Systematic Review
Source: Int J Mol Sci. 2026 Sep 26;27(19):8625. doi: 10.3390/ijms27198625 (PMC13665079; doi:10.3390/ijms27198625)
Supplement: Supplementary file 1 [file ijms-27-08625-s001.zip › Table S5. Biomarkers and Pathogenesis.pdf]

Table S5. Biomarkers and Pathogenesis

**References**	**Microbial Composition Findings**	**Vaginal pH**	**Enzymatic Activity**	**Metabolomic Findings**	**Immune & Cytokine Findings**	**Risk Factors & Progression Mechanisms**
Chao et al. (2020) [13]	High abundance *of Prevotella bivia, Enterococcus durans*, and *Porphyromonas uenonis* is observed. Low abundance of *Lactobacillus iners* and *Prevotella disiens* is noted.	Not reported	Not reported	Not reported	Not reported	*Prevotella bivia* abundance over 0.05554% combined with *Prevotella disiens* under 0.02196% acts as a predictor for CIN2+ appearance. A lack of *Lactobacillus iners* may prevent the restoration of normal vaginal microbiota, leading to persistent HR-HPV.
Chao et al. (2021) [14]	*Stenotrophomonas, Streptococcus*, and *Pseudomonas* are increased in HSIL. *Dialister, Faecalibacterium, Bifidobacterium*, and *Bacteroides* are depleted in HSIL.	Not reported	Not reported	Not reported	Faecalibacterium is identified as an immune-modulating bacterial genus.	High microbiome diversity and *Lactobacillus* depletion correlate with the severity of cervical neoplasm. Specific abundance cut-offs of *Stenotrophomonas, Faecalibacterium,* or *Bifidobacterium* can predict HSIL in HPV 16/18 infections.
Chen et al. (2020) [15]	HPV infection increases *Prevotella, Bacillus, Sneathia, Megasphaera, Streptococcus,* and *Anaerococcus.*	HPV infection alters the acidic environment of the vagina.	*Gardnerella vaginalis* produces sialidase.	Sialidase facilitates the destruction of the mucus layer. Community state type IV (CST IV) is associated with higher levels of amine production. Amine production results in the generation of carcinogenic nitrosamines.	*Sneathia* spp. may impact cellular targets such as the expression of immunosuppressive cytokines.	*Bacillus* and *Anaerococcus* proportions are positively related to the progression of CIN severity. *Gardnerella vaginalis* and its sialidase entrap anaerobic bacteria, aiding viral persistence.

Table S5. *Cont.*

References	Microbial Composition Findings	Vaginal pH	Enzymatic Activity	Metabolomic Findings	Immune & Cytokine Findings	Risk Factors & Progression Mechanisms
Cheng et al. (2020) [16]	Bacterial vaginosis-associated bacteria (BVAB) 1 and 2*, Sneathia, Prevotella, and Megasphaera* were significantly more prevalent in HPV-infected women. BVAB 1 was found almost exclusively in the vaginal microbiota of HPV-infected young women. Non-*Lactobacillus*-dominant microbiota profiles were significantly more common in HPV-infected women, while *Lactobacillus* crispatus dominance was more frequent in uninfected women.	Not reported	Not reported	Not reported	Not reported	Women aged 19–24 years had the highest risk of being infected with any HPV, oncogenic HPV, or multiple HPV types compared to women under 18. Women with non-*Lactobacillus*-dominated vaginal microbiota had more than twice the risk of having an infection with any HPV, oncogenic HPV, or non-oncogenic HPV compared to those with *L. crispatus*-dominated microbiota. HPV vaccination halved the risk of oncogenic HPV infection but demonstrated no significant influence on vaginal microbiota composition or diversity.
Dai et al. (2025) [17]	*Fannyhessea, Gardnerella, Aerococcus*, and *Streptococcus* correlate negatively with beneficial metabolites.	Not reported	Not reported	*Lactobacillus* correlates positively with D-lactic acid and DL-indole-3-lactic acid.	CIN1-enriched genes are linked to immune responses and B cell differentiation.	D-lactic acid and DL-indole-3-lactic acid protect against tumor growth and inflammation. Host differentially expressed genes (DEGs) in the cervix modulate the vaginal microbiome and metabolome across distinct CIN grades.

Table S5. *Cont.*

References	Microbial Composition Findings	Vaginal pH	Enzymatic Activity	Metabolomic Findings	Immune & Cytokine Findings	Risk Factors & Progression Mechanisms
Dou et al. (2024) [18]	*Lactobacillus* abundance is reduced in HPV infections. *Gardnerella vaginalis* prevalence is significantly increased in HPV infections.	There is no statistically significant difference in vaginal pH between HPV-positive and HPV-negative groups.	*Gardnerella* releases cytolysin and sialic acid glycosidase. HPV infection alters 13 enzymes including long-chain fatty acid CoA ligase and alpha-amylase.	Not reported	*Gardnerella* biofilms and cytolysin inhibit vaginal mucosal barrier immunoglobulin A.	Ureaplasma urealyticum parvum (Uup3 and Uup6) co-infections are significantly associated with HPV infection. The degradation of mucosal protective factors by *Gardnerella* assaults epithelial cells and increases the risk of persistent infection.
Fan et al. (2024) [19]	*Proteobacteria, Corynebacterium, Coprococcus, Peptococcus,* and *Ruminococcus* are biomarkers for cervical cancer. *Saccharopolyspora* is a biomarker for high-risk HPV.	Not reported	Not reported	Not reported	Not reported	*Proteobacteria* is significantly increased in CC compared with CIN patients; authors hypothesize it may be involved in tumor progression, noting it correlates with tumor burden in colon cancer. The diversity of vaginal species in cervical cancer patients is higher than in high-risk HPV or CIN patients.
Guo et al. (2022) [20]	HSIL is characterized by the depletion of *Lactobacillus* and accumulation of *Gardnerella, Prevotella, Fannyhessea, Megasphaera*, and *Sneathia*.	Not reported	*Prevotella* and *Gardnerella* have limited expression of degrading enzymes and are normally inhibited by *Lactobacillus*-derived hydrogen peroxide.	*Lactobacillus* produces lactic acid and bacteriocins to inhibit pathogens.	*Sneathia* spp. promotes carcinogenesis via the activation of proinflammatory pathways and inhibition of immunocytotoxicity.	Bacterial vaginosis-associated anaerobes enable persistent viral infection and subsequent disease development by impacting cellular pathways.

Table S5. *Cont.*

References	Microbial Composition Findings	Vaginal pH	Enzymatic Activity	Metabolomic Findings	Immune & Cytokine Findings	Risk Factors & Progression Mechanisms
Huang et al. (2018) [21]	*Oribacterium*, *Lachnobacterium*, and *Thermus* are unique to HPV16. *Motilibacter* is unique to HPV52. *Litorilinea* and *Paludibaculum* (with low *L. iners*) are unique to HPV58. *Coriobacteriales* and *Atopobiaceae* are enriched in 16SIL. *Burkholderia stagnalis* is highly prevalent (>94%) across cohorts.	*Lactobacillus* spp. lower environmental pH by secreting lactic acid.	Not reported	Not reported	Not reported	Specific bacterial combinations (rather than general diversity) influence the acquisition of specific hrHPV types. Synergistic bacterial networks (e.g., *Pseudomonas*, *Rhodococcus*, *Klebsiella*) increase in proportion in 16HSIL, enhancing susceptibility to HPV16 infection.
Ilhan et al. (2019) [22]	Invasive cervical carcinoma is associated with non-*Lactobacillus* dominant communities.	Vaginal pH is significantly higher in cervical cancer (pH 6.6) compared to HPV-negative controls (pH 4.9).	Gardnerella is the main contributor to hippurate metabolism.	3-hydroxybutyrate, eicosenoate, and oleate/vaccenate are strong discriminators for invasive cervical carcinoma. Non-*Lactobacillus* communities perturb amino acid and nucleotide metabolism.	Sphingolipids, plasmalogens, and linoleate positively correlate with genital inflammation markers like TNF and IL-8. Adenosine and cytosine negatively correlate with genital inflammation.	Elevated lipids act as precursors to pro-inflammatory cytokines that promote cancer development.

Table S5. *Cont.*

References	Microbial Composition Findings	Vaginal pH	Enzymatic Activity	Metabolomic Findings	Immune & Cytokine Findings	Risk Factors & Progression Mechanisms
Jung et al. (2025) [23]	*Streptococcus agalactiae, Fannyhessea vaginae,* and *Sneathia vaginalis* increase in invasive cervical cancer with HPV 16/18.	Not reported	HPV 16/18 infections show a higher abundance of mucin-degrading enzymes including sialidase, fucosidase, and galactosidase.	Not reported	The vaginal microbiome in HPV 16/18 is enriched in immune response pathways including JAK-STAT and TNF signaling.	Mucin-degrading enzymes break down cervical mucus, weakening the protective barrier and facilitating viral penetration. Stronger bacterial-fungal correlations in HPV 16/18 indicate shifts in the microbial community that impact persistence.
Kazakova et al. (2024) [24]	*Streptococcus* spp. and *Corynebacterium* spp. are more prevalent in patients with HSIL.	Not reported	*Streptococci* possess metallopeptidases capable of cleaving extracellular matrix proteins such as fibronectin and type IV collagen.	Not reported	*Streptococcal* colonisation activates multiple pro-inflammatory cytokines, including interleukin-17. *Corynebacterium* can stimulate cytokine production.	Metallopeptidases facilitate bacterial penetration into tissues and dissemination. *Corynebacterium* can adhere to epithelial cells and compete for adhesion sites, promoting dysbiosis.
Lee et al. (2013) [25]	HPV+ women exhibit significantly higher microbial diversity and lower *Lactobacillus* abundance (47% vs 77% in discordant twins). *L. iners* is significantly depleted in HPV+ twins. *Sneathia, Prevotella, Dialister, Bacillus,* and *Megasphaera* are enriched in HPV+. *Sneathia* serves as a key biomarker for high-risk HPV.	*Lactobacillus* maintains low pH. HPV16 E5 protein is susceptible to low pH. Postmenopausal status increases pH due to loss of estrogen/glycogen.	Not reported	*Lactobacillus* metabolizes epithelial glycogen into lactic acid.	*Fusobacteria* and *Sneathia* adhere to and invade epithelial cells, evading host immune surveillance, eliciting pro-inflammatory responses, and causing mucosal tissue damage.	Host genetics, menopause, and low estrogen influence microbiota. *Sneathia* and *Fusobacteria* colonisation induces mucosal immune imbalance, facilitating viral persistence and chronic inflammation.

Table S5. *Cont.*

References	Microbial Composition Findings	Vaginal pH	Enzymatic Activity	Metabolomic Findings	Immune & Cytokine Findings	Risk Factors & Progression Mechanisms
Lee et al. (2020) [26]	Identifies a *Lactobacillus* amylovorus-dominant type (CST*, 16.7%) in Asian women. Streptococcus species (*S. agalactiae, S. anginosus, S. canis, S. vestibularis, S. massilensis)* and *L. iners* are enriched in CIN2+ vs CIN1-. A 33-species Random Forest model (led by *L. iners)* predicts CIN2+ with AUC=0.952.	Not reported	*Streptococcus anginosus* causes active lysis of vaginal epithelial cells.	Not reported	Cervical microbial changes correlate with immune-related cellular recruitment.	*L. iners* dominance combined with pathobionts like *S. agalactiae* (Group B Strep) and *S. anginosus* promotes epithelial damage and tissue breakdown, accelerating progression from low-grade to high-grade CIN (CIN2+).
Lin et al. (2022) [27]	HPV+ women show decreased *Lactobacillus* (62.91% vs 95.73%) and increased *Gardnerella* (12.22%) and *Prevotella* (6.78%). HR-HPV infection rate is significantly higher in *G. vaginalis*-dominated women (72.73%) vs *Lactobacillus*-dominated women (44.72%).	CST IV features an elevated vaginal pH (>4.5).	Sialidase (SNA) positive rate is significantly higher in hrHPV+ women (44.05% vs 27.75%) and CIN patients (31.37% vs 17.46%). Catalase and leukocyte esterase show no significant association.	*Gardnerella* and *Prevotella* secrete SNA. *L. crispatus* produces H_2O_2 and lactic acid.	BV is associated with elevated IL-1β and decreased IL-17. *G. vaginalis* releases vaginolysin/cytolysin , which inhibits mucosal IgA protection.	Co-infection of *Gardnerella* and *Prevotella* represents a high-risk combination. SNA degrades mucosal protective sialoglycans and mucins, destroying the epithelial barrier, facilitating HPV entry, evading host immunity, and driving CIN progression.
Liu, Li, et al. (2023) [28]	Microbial diversity increases progressively across non-BV/HPV- > non-BV/HPV+ > BV/HPV- > BV/HPV+. 12 genera (*Gardnerella, Prevotella, Sneathia, Dialister, Fastidiosipila*, etc.) are enriched in HPV+ and further elevated in BV+. *Gardnerella, Prevotella,* and *Sneathia* peak in CIN1.	Low pH maintained by *Lactobacillus* inhibits entry of HPV and pathogens.	*Prevotella* produces sialidase, breaking down the protective mucosal layer.	*Lactobacillus* produces bacteriocins and protective organic acids.	*Prevotella* strengthens inflammatory response and immune activation. Sialidase degrades mucosal IgA and structural barriers.	BV is the strongest contributor to microbial dysbiosis. *Prevotella* and *Sneathia* act as key risk factors that alter host immune responses, promoting HPV acquisition and low-grade CIN development.

Table S5. *Cont.*

References	Microbial Composition Findings	Vaginal pH	Enzymatic Activity	Metabolomic Findings	Immune & Cytokine Findings	Risk Factors & Progression Mechanisms
Liu, Wang, et al. (2023) [29]	*Lactobacillus* and *Pseudomonas* significantly decline as CIN severity progresses (lowest in CIN3). *Gardnerella*, *Prevotella*, *Dialister*, and *Ureaplasma* increase with lesion grade. LEfSe identifies *Prevotella*, *Gardnerella, Sneathia, Megasphaera, Dialister*, and *Ureaplasma* as biomarkers for CIN3.	Depletion of *Lactobacillus* leads to elevated vaginal pH above 4.5.	Anaerobic/NLD flora increases L-lactic acid to D-lactic acid ratio, elevating extracellular matrix metalloproteinase inducers (EMMPRIN) and metalloproteinase-8 (MMP-8) activity.	High-grade CIN suppresses sugar metabolism (glycolysis, PTS, galactose/fructose metabolism) and enhances amino acid enzymes, protein folding, and fatty acid metabolism.	*Lactobacillus* maintains a localized anti-inflammatory microenvironment. High-grade CIN amplifies bacterial secretion systems.	Disruption of the balance between (*Lactobacillus* + *Pseudomonas*) vs (*Gardnerella* + *Prevotella* + *Dialister*) drives CIN. Elevated MMP-8 and EMMPRIN compromise cervical epithelial integrity, facilitating HPV anchoring, replication, and oncogenic progression.
Mitra et al. (2015) [30]	Five community state types (CSTs) were identified. CST IV (*Lactobacillus*-depleted, high diversity) prevalence increased with disease severity: 10% in normal, 21% in LSIL, 27% in HSIL, and 40% in invasive cervical cancer. Frequency of CST I (*L. crispatus*) decreased with disease severity. HSIL samples were characterized by significantly higher levels of *Sneathia sanguinegens, Anaerococcus tetradius,* and *Peptostreptococcus anaerobius.* LSIL samples had significant overrepresentation of *Lactobacillus jensenii* and *Lactobacillus coleohominis*.	Not reported.	Not reported.	Not reported.	Not reported.	Advancing CIN disease severity is strongly associated with increasing vaginal microbiota diversity and a decreasing relative abundance of *Lactobacillus* spp. A high-diversity, *Lactobacillus*-depleted microbiome (CST IV) correlates with clinically significant pre-invasive or invasive disease, potentially driving viral persistence and disease progression.

Table S5. *Cont.*

References	Microbial Composition Findings	Vaginal pH	Enzymatic Activity	Metabolomic Findings	Immune & Cytokine Findings	Risk Factors & Progression Mechanisms
Mitra et al. (2020) [41]	Baseline *Lactobacillus*-dominant VMB significantly increases 12-month CIN2 regression rates (70.0% vs 42.1%, aOR 3.56). CST IV (*Lactobacillus*-depleted, high diversity) is strongly linked to non-regression at 12 months (aOR 3.85) and 24 months (aOR 4.25). Non-regression is enriched with *Megasphaera*, BVAB1, *Prevotella timonensis*, *G. vaginalis, Sneathia,* and *Atopobium*.	*Lactobacillus* maintains low pH, supporting epithelial barrier function and inhibiting anaerobes.	Strict anaerobes produce enzymes and metabolites that compromise the mucosal barrier. *(Note: The authors broadly mention enzymes and metabolites, but do not specifically name sialidases or proteolytic enzymes in their text.)*	*Lactobacillus* produces protective lactic acid and bacteriocins. Anaerobic metabolism generates mucosal-damaging end-products.	Anaerobic taxa induce pro-inflammatory cervical cytokine production, establishing chronic inflammation that favours lesion persistence and neoplastic transformation.	Baseline depletion of *Lactobacillus* and enrichment of *Megasphaera*, *P. timonensis, G. vaginalis*, and BVAB1 drive chronic local inflammation and mucosal barrier degradation, impeding viral clearance and preventing CIN2 regression.
Morales et al. (2022) [31]	The most prevalent microbiome profiles were CST III (37.4%), CST I (30.4%), and CST IV (26.2%). 30 specific bacterial taxa were retained in a Microbiome-Based (MB) score to predict high-risk HPV (hrHPV). Vaginal colonizers associated with hrHPV dysbiosis included *Prevotella* spp., *Dialister* spp., *Megasphaera* spp., *Leptotrichia* spp., BVAB-3 (*Mageeibacillus indolicus*), and *Mycoplasma genitalium*.	More than half (52%) of the participants had an abnormal vaginal pH (>4.5).	Not reported.	Potassium hydroxide was used to check for volatile amines indicative of microbial anaerobic metabolism.	Not reported.	A Microbiome-Based (MB) score strongly correlated with hrHPV positivity (AUC = 0.8022), exceeding the predictive value of sociodemographic and behavioral variables. CST IV and bacterial vaginosis (BV) were positively associated with any HPV infection. Polymicrobial flora serves as a strong indicator of hrHPV infection and may have clinical value for risk stratification.

Table S5. *Cont.*

**References**	**Microbial Composition Findings**	**Vaginal pH**	**Enzymatic Activity**	**Metabolomic Findings**	**Immune & Cytokine Findings**	**Risk Factors & Progression Mechanisms**
Norenhag, J. (2024) [46]	Women with cervical dysplasia showed higher vaginal microbial diversity and a more dysbiotic microbiome, characterised by increased *Gardnerella* vaginalis, Aerococcus christensenii, Peptoniphilus lacrimalis, and *Fannyhessea vaginae*, while *Lactobacillus crispatus* was more abundant in healthy women. CST-IV was the predominant microbial profile among women with dysplasia (44.6%).	Not reported	G. vaginalis was the primary contributor to peptidoglycan biosynthesis and maturation pathways in women with dysplasia. *F. vaginae* was the sole species driving starch degradation V in women with dysplasia. *L. jensenii* (elevated in healthy controls) was an important driver of galactose and sucrose degradation. *Streptococcus* spp. contributed to the pentose phosphate pathway (non-oxidative branch) in the dysplasia cohort.	Women with dysplasia showed a higher abundance of 18 functional pathways, primarily involved in nucleotide biosynthesis (e.g., inosine-5'-phosphate biosynthesis) and L-alanine/L-valine biosynthesis. Healthy controls showed higher abundance of 20 functional pathways, strongly linked to amino acid biosynthesis (especially L-lysine), sugar degradation, and coenzyme A biosynthesis, largely driven by *L. crispatus*.	Not reported	The metabolic landscape in dysplasia (e.g., purine and pyrimidine biosynthesis) reflects increased energy demands, metabolic flexibility, and a focus on growth and proliferation associated with cancer cell survival. Positivity for high-risk HPV or potential high-risk HPV was significantly associated with an altered microbiota compared to low-risk or HPV-negative women. Women with high-grade squamous intra-epithelial lesions (HSIL) reported more abnormal vaginal discharge, which correlated with higher levels of dysbiotic taxa like *P. lacrimalis*, *A. christensenii*, and *G. vaginalis*.

Table S5. *Cont.*

References	Microbial Composition Findings	Vaginal pH	Enzymatic Activity	Metabolomic Findings	Immune & Cytokine Findings	Risk Factors & Progression Mechanisms
Berggrund et al. (2020) [40]	Evaluated self-sampled vaginal fluid in single HPV16-infected women. Clustering yielded two *Lactobacillus*-dominated clusters (*Lactobacillus* spp. and *L. iners*) and two non-*Lactobacillus* mixed clusters (*Gardnerella*, *Prevotella*, *Atopobium, Sneathia, Streptococcus, Aerococcus*). HPV-negative women had lower prevalence of non-*Lactobacillus* clusters (28%) vs HPV16-positive women (42%) (p=0.0173).	Not reported	Not reported	Not reported	Initial basal layer HPV infection causes minimal inflammation and evades immunity (E6 downregulates IL-1β), but persistent infection and CIN2+ development intensify local inflammation.	Women with persistent HPV16 infection and CIN2+ showed a significantly higher transition rate between microbial clusters (34%) vs those with transient HPV16 infection (19%) (p=0.036). Higher instability and dynamic fluctuations in the vaginal microenvironment favour persistent HPV16 infection and CIN2+ progression.
Pu et al. (2025) [33]	Compared cervicovaginal secretion and cervical tissue microbiomes. Tissue showed 59.2% unique species, but key dominant species overlapped *(L. iners, L. crispatus, L. jensenii, Gardnerella vaginalis, Atopobium vaginae, Prevotella bivia, Streptococcus anginosus*). *L. iners* increased post-HPV infection, then decreased with CIN formation. Tissue bacteria *P. bivia, K. pneumoniae, F. periodonticum, P. copri, V. dispar,* and *C. symbiosum* correlated with persistence/CIN.	Cervicovaginal pH was significantly higher in HPV+ (6.95± 0.77) and CIN (7.02± 1.18) vs healthy controls (5.99± 0.92, p<0.001). Tissue *P. bivia* and *Streptococcus infantis* positively correlated with pH.	*P. bivia* produces lipopolysaccharides (LPS) and invades cervical tissue.	Non-targeted metabolomics showed significant dysregulation of glycerophospholipid metabolism in CIN (e.g., PE (18:1/0:0), PI (40:6)), but no change in short-chain fatty acids (SCFAs). Specific linkages: *P. bivia*–PE (18:1/0:0)–HPV and *F. periodonticum*–PI (40:6)–HPV.	Altered glycerophospholipid metabolites and tissue microbiota interact to drive localized inflammatory responses.	Higher BMI and elevated pH correlate with microbial shifts. CIN primarily alters the cervical tissue microenvironment (beta diversity changed in tissue, not secretion). *P. bivia* and *F. periodonticum* interplay with altered lipid metabolites to sustain tissue inflammation, promoting HPV persistence and CIN progression.

Table S5. *Cont.*

References	Microbial Composition Findings	Vaginal pH	Enzymatic Activity	Metabolomic Findings	Immune & Cytokine Findings	Risk Factors & Progression Mechanisms
Santella et al. (2022) [34]	Main phyla: *Firmicutes, Bacteroidetes, Proteobacteria, Actinobacteria. Firmicutes* decreased in HPV+ (87.4% vs 90.7%), while *Proteobacteria, Actinobacteria,* and *Bacteroidetes* increased. HPV+ women showed higher abundance of *Enterococcus* (6.9%), *Staphylococcus* (4.2%), *Gardnerella* (1.7%), *Morganella* (1*%), E. faecalis, S. haemolyticus, L. acidophilus,* and *L. iners.* Controls dominated by *L. crispatus, L. iners, L. taiwanensis.*	*Lactobacillus* spp. maintains low pH via lactic acid and H_2O_2 production. Low pH increases cervical mucus viscosity, trapping viral particles and protecting the epithelial barrier.	*Lactobacillus* produces glycogen-fermenting enzymes.	Lactic acid and hydrogen peroxide (H_2O_2) production by *Lactobacillus spp.*	*Staphylococcus aureus* secretes toxins and immune-modulating factors. Disrupted microbiota alters local immune surveillance and host mucosal barrier integrity.	High microbial diversity, depletion of *Lactobacillus* spp., and overgrowth of anaerobes/pathobionts (*Gardnerella, Morganella, Staphylococcus, Enterococcus*) reduce cervical mucus viscosity, impairing viral trapping and enabling HPV infection, persistence, and progression to CIN.
Shi (2022) [42]	73 women with single HR-HPV (HPV16/52/58). CSTs identified: CST I (*L. crispatus*), CST II, CST III (*L. iners*), CST IV (diverse), CST V. Depletion of Enterococcus ASV_62 and high baseline abundance of *L. iners* significantly associated with failure to clear HR-HPV at 12 months (log-fold change = 4.16, q<0.001).	Not explicitly measured.	*L. iners* encodes inerolysin (a cholesterol-dependent cytolysin) which damages host cell membranes.	*L. iners* produces low amounts of lactic acid and lacks synthesis genes for host-protective bacteriocins compared to *L. crispatus.*	*L. iners* is positively correlated with pro-inflammatory cytokines in vaginal fluid.	High relative abundance *of L. iners* at baseline is an independent risk factor for failure of HR-HPV clearance at 12 months (aOR = 3.34, p=0.044), particularly in patients under non-operative management (aOR = 3.94, p=0.041). *L. iners* flexibility, inerolysin production, lack of protective peptides/D-lactic acid, and induction of inflammation favor viral persistence.

Table S5. *Cont.*

References	Microbial Composition Findings	Vaginal pH	Enzymatic Activity	Metabolomic Findings	Immune & Cytokine Findings	Risk Factors & Progression Mechanisms
So et al. (2020) [35]	Marked decrease of *L. crispatus* in CIN/cancer vs normal (p=0.022). Diversity increased with disease grade. Species significantly associated with risk of CIN2/3 or cervical cancer: *Atopobium vaginae* (OR 4.33), *Dialister invisus* (OR 4.89), *Finegoldia magna* (OR 6.00), *Gardnerella vaginalis* (OR 7.43), *Prevotella buccalis* (OR 11.00), and *Prevotella timonensis* (OR 6.00). Also enriched: *Peptostreptococcus anaerobius, Porphyromonas uenonis.*	Predominant *Lactobacillus* spp. maintains low pH, creating a chemical barrier. Elevated pH is associated with cervical carcinogenesis.	Low-*Lactobacillus* microenvironment upregulates mucin-degrading enzymes and downregulates H_2O_2 production.	Production of mucin-degrading enzymes and loss of protective H_2O_2.	Anaerobic co-infections aggravate cervical mucosal inflammation, dysregulate cytokine production, and compromise epithelial barrier function.	Depletion of *L. crispatus* combined with an overgrowth of obligate/facultative anaerobes (*G. vaginalis, A. vaginae, Prevotella spp., D. invisus, F. magna*) disrupts mucosal barriers and elevates mucin degradation, directly driving persistent HPV infection and neoplastic progression to CIN2/3 and invasive cancer.
Tossas et al. (2023) [43]	Cohort of 3,050 women (70% non-Latina [nL] Black, 30% nL White). Grouped into Optimal (*L. crispatus, L. gasseri, L. jensenii*), Moderate (*L. iners*), and Suboptimal (*G. vaginalis, A. vaginae, Ca. Lachnocurva vaginae*). nL Black women had higher suboptimal VMB (58% vs 38%). *L. crispatus* significantly depleted in women developing CIN3.	Optimal VMB associated with low pH and anti-inflammatory properties.	Not reported	Not reported	Suboptimal VMB triggers local inflammation and immune dysregulation. Chronic stress in nL Black women may contribute pro-inflammatory stimuli.	Race modifies the VMB–CIN3 association (p=0.04). Suboptimal VMB increases crude CIN3 risk 3-fold (OR = 2.91). In nL Whites, suboptimal VMB significantly increased CIN3 risk vs optimal VMB (OR = 6.0, p=0.02). Surprisingly, optimal VMB was NOT protective in nL Black women: nL Black women with optimal VMB had a 7.8-fold higher risk of CIN3 compared to nL White women with optimal VMB (p=0.007), highlighting host/environmental stress interactions.

Table S5. *Cont.*

References	Microbial Composition Findings	Vaginal pH	Enzymatic Activity	Metabolomic Findings	Immune & Cytokine Findings	Risk Factors & Progression Mechanisms
Usyk et al. (2020) [44]	At Visit 1 (V1), *L. iners* associated with clearance (LDA > 4.0), while *Gardnerella* was the top biomarker for CIN2+ progression (LDA > 4.0). At Visit 2 (V2), *Prevotella amnii, Porphyromonas circumdentaria, Parvimonas micra, Anaerococcus prevotii* enriched in progression. Fungi: *Candida albicans* dominant.	*Lactobacillus* dominance maintains low pH.	Imputed bacterial Cell Motility pathway protective against progression (OR=0.75, p= 0.019).	Imputed pathways: Xenobiotics Biodegradation positively associated with progression (p=0.002); Cell Motility pathway protective (OR=0.75).	Bacterial flagella (Cell Motility pathway) activate host mucosal innate/acquired immunity to aid viral clearance. *Gardnerella* exerts immunosuppressive effects in the cervicovaginal niche (e.g., reducing IL-17).	Multivariate GLM: V1 *Lactobacillus* (OR=0.41), V1 fungal diversity (OR=0.90), and V1 Cell Motility (OR=0.75) protect against CIN2+; V2 bacterial diversity increases CIN2+ risk (OR=1.17). Mediation analysis proved *Gardnerella* at V1 does not directly cause CIN2+ but acts by inducing elevated polymicrobial diversity at V2 (p=0.040), which drives progression to CIN2+.
Xu et al. (2023) [36]	VMB diversity is significantly higher in HRCD (p<0.05). *Firmicutes* lower in HRCD (43.7% vs 57.9%); *Bacteroidetes, Epsilonbacteraeota, Cyanobacteria* higher. *Lactobacillus* is significantly lower in HRCD (p<0.01). *Halomonas, Porphyromonas, Bacteroides, Peptoniphilus, Anaerococcus, Ezakiella, Fusobacterium, Enterococcus* enriched in HRCD. LEfSe biomarkers for HRCD: *Clostridia, Fusobacterium, Enterococcus, Streptococcus agalactiae*.	Not reported	ABC transporters and phosphotransferase system (PTS) enriched in HCs; nitrogen, methane, and oxidative phosphorylation pathways enriched in HRCD.	Healthy controls enriched in carbohydrate metabolism (glycolysis, fructose/mannose, galactose) and ABC transporters. HRCD is enriched in cofactor/vitamin metabolism, porphyrin/chlorophyll metabolism, terpenoids/polyketides, nitrogen, and oxidative phosphorylation. High-diversity microbiota utilizes amino acids/nitrogen as primary energy sources over glycogen.	HPV E7 oncoprotein suppresses host defense peptides via NF-κB and Wnt/β-catenin pathways, impairing *Lactobacillus* survival. *Peptostreptococcus anaerobius* and *Fusobacterium* skew macrophage polarisation and disrupt mucosal homeostasis.	Depletion of *Lactobacillus* and overgrowth of anaerobes (*Fusobacterium*, *Enterococcus, S. agalactiae, Ezakiella*) leads to ectopic nitrogen/lipid metabolism, suppression of mucosal defense peptides by HPV E7, macrophage skewing, and progression to CIN and cervical cancer.

Table S5. *Cont.*

References	Microbial Composition Findings	Vaginal pH	Enzymatic Activity	Metabolomic Findings	Immune & Cytokine Findings	Risk Factors & Progression Mechanisms
Yang et al. (2025) [37]	173 Taiwanese women (HPV-, HPV+ w/o neoplasia, CIN1, CIN2+). VALENCIA CST IV sub-types dominated by *Staphylococcus* (CST IV-C4, 27.2%), *Streptococcus* (CST IV-C1, 18.4%), *Enterococcus* (CST IV-C2, 13.2%), *G. vaginalis / F. vaginae* (CST IV-B, 2.9%). *Enterococcus faecalis* increases with CIN severity.	*Lactobacillus* maintains acidity*; L. iners* produces L-lactic acid which is less protective than D-lactic acid.	Vaginal *Porphyromonas* species (*P. asaccharolytica, P. uenonis*) display dose-dependent secreted proteolytic activity that degrades host extracellular matrix and coagulation factors.	Proteolytic degradation of host proteins by BV-associated anaerobes serves as a primary virulence mechanism.	*Sneathia vaginalis* adheres to malignant cervical cells and releases toxic products. *S. aureus* secretes toxins and immune-modulating factors. *S. pyogenes* causes chronic inflammation, aiding viral persistence.	Depletion of D-lactic acid-producing Lactobacilli + overgrowth of CST IV sub-communities (*Staphylococcus, Streptococcus, Sneathia, Porphyromonas, Fannyhessea*). Proteolytic matrix breakdown by *Porphyromonas* and toxic cell adhesion by *S. vaginalis* impair immune clearance, facilitating persistent HPV infection and driving CIN1 to CIN2+ progression.
	Biomarkers*: Fannyhessea vaginae* strongly linked to HPV+ (p=0.032, PPV = 74.2%); *Phocaeicola vulgatus* linked to HPV-; *Sneathia vaginalis, Prevotella intermedia, Rothia amarae* enriched in HPV+ without CIN; *Aerococcus christensenii* biomarker for CIN2+. *Streptococcus (S. pyogenes)* associated with alpha-7 HPV types (18, 45, 70); *Staphylococcus (S. haemolyticus)* associated with 9 HPV types (16, 31, 52, 58)					

Table S5. *Cont.*

References	Microbial Composition Findings	Vaginal pH	Enzymatic Activity	Metabolomic Findings	Immune & Cytokine Findings	Risk Factors & Progression Mechanisms
Zeng et al. (2023) [45]	90 HPV+ patients, 45 controls. hrHPV16/18 group showed significantly higher alpha diversity vs controls ($p<0.01$). Dominant species in HPV+: *L. iners* (54.44%), *S. amnii* (11.11%). Top species in persistent HPV: *L. iners* (53.13%), *S. amnii* (14.06%), *L. delbrueckii* (4.69%). Persistent HPV group showed significantly higher levels of *Bacteroidaceae, Erysipelotrichaceae, Helicobacteraceae, Neisseriaceae, Streptococcaceae* and *Fusobacterium, Bacteroides, Neisseria, Helicobacter* vs cleared HPV ($p<0.05$). Also higher*: S. amnii, E. faecalis, S. haemolyticus, S. intermedius, P. amnii, P. timonensis.*	Decrease in protective Lactobacilli leads to increased vaginal pH, weakening pathogen defense and damaging mucosal barriers.	BV-associated bacteria increase epithelial lining-degrading enzymes.	*L. iners* produces low lactic acid and lacks genes for bacteriocin synthesis.	*Fusobacterium* predominance in cervical microenvironment promotes expression of IL-4 and TGF-β1 mRNA, inducing an immunosuppressive phenotype (activating Treg cells and dendritic cells).	Infection with Hr-HPV16/18 or multiple HPV types + *L. iners* dominance or enrichment of pathobionts (*S. amnii, Fusobacterium, Bacteroides, Neisseria, Helicobacter, E. faecalis, P. timonensis*) creates an immunosuppressive local microenvironment (via IL-4/TGF-β1), elevates pH, and impairs host immune clearance, causing persistent HPV infection. Addition of vaginal *Lactobacillus* probiotics to interferon therapy enhances viral clearance.
Zhang et al. (2018) [38]	Firmicutes, Actinobacteria, and Proteobacteria were the dominant phyla overall. Lactobacillus iners and L. crispatus were predominant in most women. HPV-positive samples were enriched with Streptococcus agalactiae, Bacteroides fragilis, Pseudomonas stutzeri, and Peptostreptococcus anaerobius. HPV-negative samples were enriched with Lactobacillus delbrueckii. CIN2+ cases showed higher abundance of L. crispatus, S. agalactiae, B. fragilis, and Campylobacter ureolyticus. CIN1- cases had higher Photobacterium damselae, L. jensenii, and Atopobium vaginae.	Not reported.	Not reported.	Not reported.	Not reported.	Cervical microbiota have direct and indirect effects on CIN progression. Pseudomonas stutzeri, Bacteroides fragilis, Lactobacillus delbrueckii, Atopobium vaginae, and Streptococcus agalactiae show indirect effects on CIN mediated by altering the natural history of HPV infection. The directions of the direct and indirect associations for Ps. stutzeri were opposite, while for A. vaginae they were the same.

Table S5. *Cont.*

References	Microbial Composition Findings	Vaginal pH	Enzymatic Activity	Metabolomic Findings	Immune & Cytokine Findings	Risk Factors & Progression Mechanisms
Zhang et al. (2022) [39]	Microbial alpha diversity was significantly higher in HPV-infected and CIN-diagnosed groups than in healthy controls. Lactobacillus crispatus decreased while Lactobacillus iners and non-Lactobacillus species increased in HPV and CIN groups. Streptococcus, Prevotella, and Chlamydia were significantly more prevalent in HPV-infected women. L. gasseri-dominated (CST-II) and L. jensenii-dominated (CST-V) clusters were significantly absent in this Chinese cohort. Proportions of CST-III and CST-IV-B increased with disease progression.	Not reported.	Not reported.	Not reported.	HPV E7 oncoprotein suppresses host defense peptides via NF-κB and Wnt/β-catenin pathways, which impairs Lactobacillus survival (noted as a mechanism).	Age is a significant risk factor for HPV infection. With increasing age, the probability of HPV infection and the proportion of the highly diverse CST-IV-B increase. The lack of L. gasseri in Chinese women may explain delayed HPV clearance and favor viral persistence.

Note: Reference numbers in this document correspond to the reference list in the main manuscript.
